# Nanomaterial-based total analysis systems for isolation and detection of exosomal biomarkers in cancer diagnosis

**DOI:** 10.1016/j.mtbio.2026.102866

**Published:** 2026-01-28

**Authors:** Myeong-Jun Lee, Minkyu Shin, Sangeun Lee, Shan Liu, Sang-Nam Lee, Jeong-Woo Choi

**Affiliations:** a Department of Chemical & Biomolecular Engineering, Sogang University, Seoul, Republic of Korea; b Department of Chemical Engineering, Research Center of Chemical Technology, Hankyong National University, Gyeonggi–do, Republic of Korea; c Sichuan Provincial Key Laboratory for Human Disease Gene Study, Department of Medical Genetics, Sichuan Academy of Medical Sciences & Sichuan Provincial People's Hospital, University of Electronic Science and Technology of China, Chengdu, China; d Uniance Gene Inc., Seoul, Republic of Korea

**Keywords:** Nanotechnology, Exosome, Cancer, Total analysis system, Isolation, Detection

## Abstract

Exosomes are nanoscale extracellular vesicles secreted by both cancerous and healthy cells that carry a diverse array of biomolecules, including nucleic acids and proteins, reflecting the physiological and pathological states of their cells of origin. This makes them highly promising biomarkers for liquid biopsy-based cancer diagnostics. However, conventional methods for exosome analysis from isolation to detection are often time-consuming, labor-intensive, and lack the sensitivity and specificity required for clinical applications. To address these limitations, nanomaterials are increasingly recognized as powerful tools for both exosome isolation and biomarker detection. Their unique physicochemical properties enable enhanced capture efficiency, precise molecular recognition, and signal amplification. In recent years, there has been increasing interest in developing nanomaterial-based total analysis systems (TAS) that seamlessly integrate exosome isolation and detection into unified, high-throughput diagnostic platforms. Consolidating the fragmented workflow into compact, high-throughput devices, these TAS hold strong promise for clinical and point-of-care (POC) applications. While previous reviews have primarily focused on either isolation or detection strategies, this work provides comprehensive overview dedicated to integrated nanomaterial-based TAS that combines both isolation and detection in a single platform. In this review, we discuss the biological relevance of exosome proteins and miRNAs as cancer biomarkers. We then examine nanomaterial-assisted strategies for exosome isolation and for signal transduction and amplification in exosome biomarker detection. Finally, we highlight newly developed TAS for exosome analysis, most reported in studies published between 2023 and 2025, emphasizing their potential for clinical application and POC applications. This review aims to establish a roadmap for future innovations in exosome-based diagnostics enabled by nanotechnology. To this end, we critically assess the current challenges and outline future directions, providing perspectives for their clinical translation.

## Graphical abstract

**Figure:**
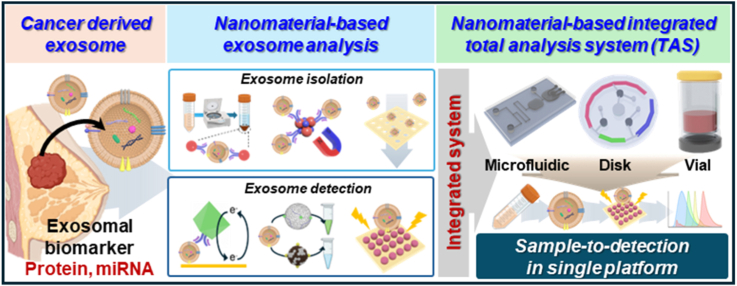

## 1 Introduction

Early and accurate cancer detection is critical for improving treatment outcomes, enabling timely interventions, and reducing patient mortality [1,2]. In recent years, liquid biopsy has become a powerful alternative to traditional tissue biopsies, offering minimally invasive access to clinically informative biomarkers [3]. Among the various analytes explored for liquid biopsy, exosomes—30 to 150 nm extracellular vesicles secreted by nearly all cell types—have recently gained considerable attention [4,5]. These vesicles encapsulate and protect a diverse repertoire of biomolecules, including proteins, lipids, DNA, and RNA, particularly microRNAs (miRNAs), which are implicated in cancer initiation, progression, and metastasis [6]. Their stability in biofluids and tumor-specific cargo profiles make exosomes highly promising candidates for non-invasive cancer diagnostics and real-time disease monitoring [7,8].

Despite their promising potential, conventional workflows for exosome-based biomarker detection remain technically challenging, time-consuming, and fragmented. Current methods typically involve sequential steps: exosome isolation/enrichment by ultracentrifugation or size-exclusion chromatography, RNA extraction and purification, enzymatic amplification (e.g., RT-qPCR), and target quantification [9]. Each step often requires separate instruments, reagents, and skilled operators, which introduces cumulative error, increases assay cost and time, and hinders point-of-care (POC) applicability [10]. Moreover, the low abundance of exosomes in early-stage cancer samples, batch-to-batch variability in exosome isolation yields, and the susceptibility of miRNAs to degradation further complicate reliable biomarker analysis [11]. These limitations underscore the urgent need for novel diagnostic platforms that streamline exosome isolation and analysis with enhanced sensitivity, reproducibility, and clinical accessibility [12].

In this context, nanomaterials have garnered increasing interest as promising tools to address these technological bottlenecks. Their unique physicochemical properties, including high surface-to-volume ratios, tunable surface chemistry, and multifunctional optical, electrical, and magnetic characteristics, enable the development of integrated platforms capable of isolating exosomes and detecting their molecular contents with high precision [[13], [14], [15]]. For example, magnetic nanoparticles (MNPs) functionalized with exosome-specific ligands have facilitated rapid and selective enrichment of exosomes from complex biological fluids [16]. Similarly, gold nanoparticles (AuNPs), quantum dots (QDs), and carbon-based nanostructures have been exploited for signal transduction and amplification in biosensing assays [17,18]. Although these nanomaterial-based approaches represent significant progress toward effective exosome analysis, most target either the isolation or the detection stage and therefore still require intermediate processing steps or sample transfers, often with limited sensitivity [19].

To address this limitation, recent advancements have focused on total analysis systems (TAS), which are fully integrated platforms that consolidate sample processing, target amplification, and signal readout within a single device or reaction unit [20]. When combined with nanomaterial-based components, these systems offer remarkable improvements in analytical performance, user convenience, and compatibility with POC settings [21]. Such platforms not only minimize sample loss and contamination by eliminating transfer steps but also enable multi-analyte detection, low-volume processing, and rapid turnaround times. Nevertheless, despite these promising developments, a comprehensive understanding of how nanomaterials contribute to each analytical stage—from exosome isolation to miRNA detection—remains limited [22].

While previous reviews, including Fang et al. or Hu et al., have comprehensively summarized nanomaterials-assisted exosome isolation and analysis techniques, they have primarily focused on individual functional components or sensing modalities [23,24]. In contrast, the present review is specifically dedicated to integrated nanomaterial-based total analysis systems (TAS), emphasizing end-to-end architectures that unify exosome isolation, signal amplification, and detection within a single, coherent workflow. By systematically categorizing microfluidic-, disk-, and vial-type TAS and evaluating the depth of nanomaterial-enabled integration, this review provides a system-level perspective that has not been explicitly addressed in prior literature.

**Fig. 1:**
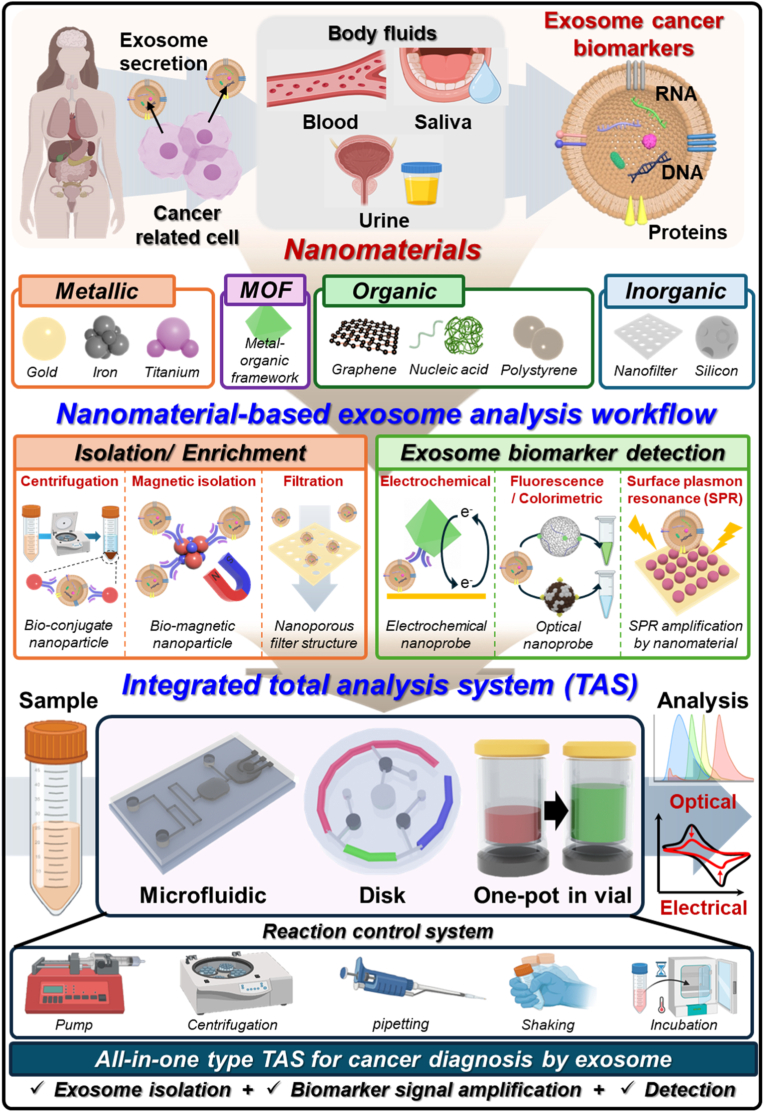
Schematic overview of nanomaterial-based total analysis systems (TAS) for exosomal biomarker detection in cancer diagnosis: Cancer-related cells secrete exosomes containing diverse molecular biomarkers (RNA, DNA, proteins) into body fluids such as blood, saliva, and urine. Various classes of nanomaterials, including metallic (gold, iron, titanium), metal-organic frameworks (MOFs), organic (e.g., graphene, nucleic acids, polystyrene), and inorganic (nanofilter, silicon) have been engineered to improve exosome isolation, enrichment, and signal transduction. The nanomaterial-based exosome analysis workflow comprises (i) isolation/enrichment via centrifugation, magnetic separation, or nanoporous filtration, and (ii) biomarker detection using electrochemical, fluorescence/colorimetric, or surface-plasmon-resonance (SPR) platforms. These modular strategies are further integrated into TAS such as microfluidic chips, disk-based platforms, and one-pot vial systems, enabling all-in-one optical or electrical analyses for rapid, sensitive, and user-friendly cancer diagnosis through exosome profiling.

In this review, we provide a comprehensive overview of nanomaterial-enabled strategies for detecting exosomal biomarkers in cancer diagnostics (Fig. 1). We first highlight the clinical relevance of exosomal proteins and miRNAs, followed by a critical assessment of nanomaterial-assisted exosome isolation techniques. We then detail recent innovations in nanomaterial-based biosensing mechanisms, including signal transduction, amplification, and platform integration. Finally, we introduce and compare different types of nanomaterial-based TAS platforms, including microfluidic, DISK-type, and vial-type, discussing their design principles, operational advantages, and translational potential. By highlighting recent advances and outlining future directions, this review aims to guide the development of next-generation exosome analysis tools for clinical and translational oncology.

## 2 Exosomes as cancer biomarkers

**Fig. 2:**
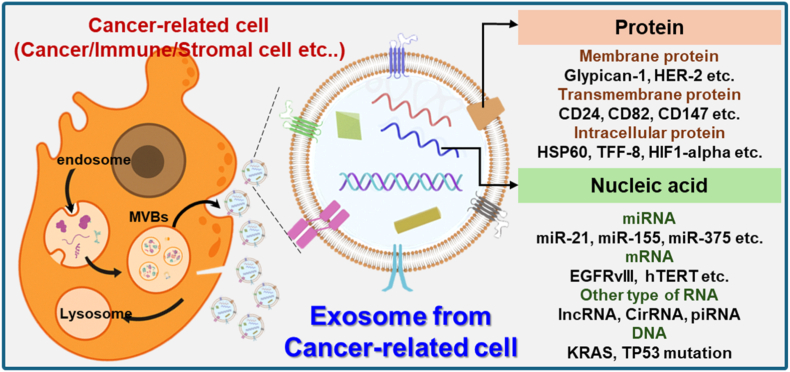
Biogenesis of exosomes and cancer diagnosis biomarkers in exosomes.

Exosomes are nanosized extracellular vesicles (EVs) actively secreted by most eukaryotic cells [25]. They are formed via the endolysosomal pathway through inward budding of multivesicular bodies (MVBs), which subsequently fuse with the plasma membrane and release their intraluminal vesicles into the extracellular space [26,27]. Unlike other EVs, such as microvesicles or apoptotic bodies, exosomes are characterized not only by their size and biogenesis but also by their enriched cargo of proteins, nucleic acids, lipids, and metabolites selectively packaged from the parent cell (Fig. 2) [[28], [29], [30]].

In the context of cancer, tumor-derived exosomes (TDEs) are critical mediators of intercellular communication, modulating processes such as immune evasion, angiogenesis, metastasis, and drug resistance [31,32]. TDEs reflect the molecular landscape of their cells of origin, making them valuable reservoirs of cancer-associated biomarkers. Their presence in virtually all bodily fluids—including blood, urine, saliva, cerebrospinal fluid, and pleural effusions—along with their inherent molecular stability provided by a protective lipid bilayer, makes them highly attractive targets for liquid biopsy [33]. Compared with conventional tissue biopsy, exosome-based diagnostics offer significant advantages, including minimal invasiveness, the ability to perform longitudinal monitoring, and the potential to capture tumor heterogeneity [34].

Although tumor-derived exosomes have been extensively investigated, increasing evidence indicates that exosomes released from non-tumor cells—such as immune, stromal, and endothelial cells—also contribute valuable information for the early detection of cancer. These vesicles reflect the dynamic interactions between tumors and their microenvironment even before a malignant lesion becomes clinically detectable. Immune-cell–derived exosomes can mirror early inflammatory or immunosuppressive changes, whereas stromal- and epithelial-cell exosomes undergo characteristic molecular alterations during precancerous remodeling [35]. Stromal-cell–derived exosomes, particularly those released from cancer-associated fibroblasts (CAFs), actively remodel the extracellular matrix and transmit metabolic and paracrine signals to neighboring epithelial and immune cells, creating microenvironmental alterations that can be detected as early indicators of tumorigenesis [36]. Endothelial-cell exosomes are also involved in the initiation of angiogenic signaling that precedes measurable tumor vascularization [37]. Together, these non-tumor-cell–derived exosomes serve as indirect yet sensitive indicators of early tumor-associated changes, complementing tumor-derived exosomal biomarkers in liquid-biopsy–based early cancer diagnosis.

Despite these advantages, the clinical application of exosomes has been hampered by several challenges. These include their low abundance in early-stage disease, overlapping physical properties with other extracellular vesicles or plasma contaminants, and the absence of standardized isolation and analysis protocols [38]. Nevertheless, a growing body of evidence supports the diagnostic and prognostic value of specific exosomal biomolecules, particularly proteins and miRNAs, as discussed in the following subsections [39,40].

### 2.1 Exosomal proteins as cancer biomarkers

Exosomes are enriched with proteins that closely reflect the molecular landscape of their originating tumor cells. The lipid bilayer membrane of these vesicles protects their protein cargo from enzymatic degradation, ensuring stability in diverse biofluids such as serum, plasma, urine, and ascites. This stability, combined with selective protein packaging mediated by ESCRT complexes, tetraspanins, and lipid raft-dependent sorting pathways, makes exosomal proteins particularly promising biomarkers for cancer detection and monitoring [41,42]. Unlike soluble proteins, which may lack specificity, exosomal proteins preserve contextual cues of tumor biology, including receptor activation states and adhesion molecule profiles, thereby providing insights not only into tumor presence but also into progression, metastasis, and therapeutic response.

In breast cancer, glypican-1 (GPC1) has been identified as a notable exosomal biomarker. In addition to its role in pancreatic cancer, recent studies have demonstrated GPC1 overexpression across different breast cancer subtypes, suggesting that this heparan sulfate proteoglycan represents a common hallmark of malignant transformation in breast tissue [43]. The detection of GPC1-positive exosomes in circulation highlights their potential for early diagnosis and stratification of breast cancer patients. Similarly, HER2, a receptor tyrosine kinase that drives tumor proliferation, has been consistently detected on exosomes derived from HER2-positive breast cancers. Importantly, HER2-bearing exosomes can bind and sequester trastuzumab, reducing the therapeutic antibody's bioavailability and thereby contributing to drug resistance [44]. Monitoring exosomal HER2 levels may thus provide predictive information on treatment outcomes while simultaneously serving as a disease-specific diagnostic marker. Furthermore, CD24 and CD82 have been identified in breast cancer exosomes, where their elevated abundance correlates with disease progression. CD24 functions as a broadly distributed exosomal marker, while CD82, a tetraspanin, has been associated with malignant progression and may provide complementary diagnostic value for monitoring tumor dynamics [45].

Exosomal proteins also serve as critical markers in other cancer types. In ovarian carcinoma, exosomes isolated from malignant ascites consistently harbor EpCAM and CD24, which together provide strong immunoaffinity handles for exosome capture and improve diagnostic specificity [46]. In colorectal cancer, exosomes enriched with CD147 have been identified as non-invasive biomarkers for both diagnosis and prognosis. Mechanistically, CD147 promotes matrix metalloproteinase activation and stromal remodeling, directly linking its exosomal abundance to invasive tumor behavior [47].

Oncogenic receptor proteins have similarly been recognized as exosomal biomarkers with significant diagnostic implications. In lung cancer, EGFR is localized on exosome membranes, and its detection in patient serum demonstrates superior discriminatory power compared with soluble EGFR assays [48]. In glioblastoma, exosomes containing the mutant receptor EGFRvIII have been shown to remodel the proteome of extracellular vesicles and propagate oncogenic signaling to recipient cells, embedding molecular signatures of tumor aggressiveness into circulating biomarkers [49]. These findings highlight the ability of exosomal proteins to reflect not only tumor presence but also the mutational and signaling states of cancer cells.

Further examples underscore the translational versatility of exosomal protein biomarkers. In prostate cancer, urinary exosomes carrying PSA represent one of the earliest demonstrations of exosome-based diagnostics, reflecting direct glandular shedding into urine and offering a non-invasive alternative to serum PSA testing [50]. Additionally, exosomal integrins (e.g., αvβ5, α6β4) have been implicated in organ-specific metastasis, as they mediate exosome uptake by stromal cells in target tissues such as the liver and lung. These integrin-mediated interactions drive pre-metastatic niche formation, providing not only mechanistic insight into metastatic dissemination but also opportunities for predictive biomarker development.

Not only the cancer cells-derived exosomes, cancer-related cells also secreted exosomal protein biomarkers for cancer diagnosis. For example, Exosomal PD-L1 released from activated macrophages and T cells binds PD-1 on cytotoxic T lymphocytes, reflecting immune suppression and correlating with tumor burden in early-stage melanoma and NSCLC. Its plasma exosomal level serves as a predictive marker for immunotherapy response and early disease progression. [51]. Exosomes secreted by dendritic cells express antigen-presenting molecules such as MHC II and CD86, which can be detected in plasma during early antitumor immune activation. Their elevation indicates host immune recognition preceding visible tumor growth [52]. Cancer-associated fibroblast (CAF)–derived exosomes enriched with QSOX1 are detectable in serum and distinguish colorectal-cancer patients from healthy controls, showing promise for early, non-invasive diagnosis [53]. Tenascin-C and periostin which are proteins secreted through fibroblast-derived exosomes participate in extracellular-matrix remodeling and are up-regulated in the circulation of patients with premalignant lesions, marking stromal activation during tumor initiation [54]. In endothelial cell derived exosomes, Developmental endothelial locus-1 (DEL-1) protein can be a good candidate for cancer diagnosis. DEL-1-positive small EVs from endothelial cells are significantly elevated in early-stage breast-cancer plasma and decrease after surgical removal for early detection [55]. Also, endothelial-derived exosomes enriched with VEGF-A and angiopoietin-2 (angiogenic proteins) are increased in the plasma of colorectal- and hepatocellular-carcinoma patients prior to measurable angiogenesis, indicating their potential as early vascular biomarkers [56].

### 2.2 Exosomal miRNAs as cancer biomarkers

Exosomal miRNAs are among the most extensively studied classes of exosomal biomarkers due to their remarkable stability, accessibility via liquid biopsy, and functional involvement in tumor biology [57]. Packaged into exosomes through selective RNA-sorting mechanisms, such as interactions with RNA-binding proteins (hnRNPA2B1, YBX1) or miRNA motif–dependent recognition, these small non-coding RNAs are protected from RNase degradation in circulation. As a result, they can be reliably detected in serum, plasma, urine, ascites, and cerebrospinal fluid. Beyond their diagnostic value, exosomal miRNAs influence recipient cells by reprogramming gene expression networks, thereby affecting proliferation, invasion, angiogenesis, drug resistance, and metastatic niche formation. Their dual role as biomarkers and functional mediators of cancer progression underscores their clinical significance.

In breast cancer, exosomal miR-21 is consistently reported as a reliable indicator. Elevated levels of miR-21 in plasma exosomes are associated with poor prognosis, enhanced cell proliferation, and suppression of tumor suppressors such as PTEN and PDCD4 [58]. The oncogenic activity of exosomal miR-21 extends to remodeling the tumor microenvironment by activating fibroblasts and promoting angiogenesis, reinforcing its role as both a mechanistic effector and a diagnostic marker. Additionally, miR-1246 has been identified as a potent driver of malignancy in breast cancer. Functionally, miR-1246 downregulates CCNG2, thereby promoting cell cycle progression, drug resistance, and invasion. Detection of miR-1246 in circulating exosomes therefore provides a clinically relevant indicator of aggressive breast cancer phenotypes and therapy resistance.

In lung cancer, dysregulation of exosomal miRNAs provides important diagnostic information. For example, miR-126 is downregulated in patient-derived plasma exosomes, reflecting impaired angiogenic signaling, while miR-21 is simultaneously upregulated, promoting oncogenic transformation [59]. The contrasting expression of these two miRNAs enables the construction of a biomarker panel with improved sensitivity and specificity compared with single-analyte assays. These alterations not only assist in diagnosis but also correlate with disease stage and progression, highlighting their prognostic utility.

In colorectal cancer (CRC), a broad spectrum of exosomal miRNAs is enriched, including let-7a, miR-1229, miR-1246, miR-150, miR-21, miR-223, and miR-23a. These miRNAs regulate pathways involved in cell adhesion, proliferation, and immune evasion [60]. Clinical studies have demonstrated that this miRNA panel can distinguish CRC patients from healthy controls with high accuracy, making it a strong candidate for early screening. Moreover, multiplexed detection of these miRNAs enhances analytical robustness and diagnostic reliability by compensating for biological and temporal variations in single-marker expression. This combinatorial approach minimizes false-positive or false-negative results that may occur when relying on a single exosomal biomarker, thereby improving the overall accuracy of CRC liquid-biopsy screening.

In pancreatic cancer, exosomal miR-196a and miR-1246 have been highlighted as early diagnostic indicators. Their elevated levels in plasma exosomes correlate with localized tumor stages, suggesting their utility for detecting pancreatic cancer before widespread metastasis. Mechanistically, miR-196a contributes to tumor progression by targeting annexin A1, while miR-1246 modulates apoptosis-related genes, collectively promoting tumor growth and survival [51].

In prostate cancer, exosomal miR-141 and miR-375 are consistently enriched in patient plasma. These miRNAs are downstream targets of androgen receptor signaling and correlate with tumor progression, metastasis, and castration-resistant prostate cancer phenotypes [61]. Detection of these miRNAs in exosomes provides a non-invasive approach to stratify patients according to disease stage and therapeutic response.

Ovarian cancer is characterized by exosomes enriched with a panel of miRNAs, including miR-21, miR-141, the miR-200 family (miR-200a, −200b, −200c), miR-203, miR-205, and miR-214 [62]. These miRNAs collectively regulate EMT, angiogenesis, and metastatic niche formation. For instance, exosomal miR-141 activates stromal fibroblasts to promote tumor invasion, while the miR-200 family suppresses ZEB1/2 to regulate epithelial plasticity. Elevated levels of these miRNAs in circulation serve as strong indicators of ovarian tumor presence and progression, while also providing mechanistic insights into metastasis.

In gastric cancer, exosomal miR-423–5p directly targets SUFU, a negative regulator of Hedgehog signaling, thereby promoting tumor growth and metastasis [63]. Its presence in serum exosomes offers a mechanistic biomarker that is both diagnostically relevant and indicative of underlying oncogenic signaling dysregulation.

In glioblastoma (GBM), multiple exosomal miRNAs, including miR-9, miR-29b, miR-34b-3p, and miR-222, are upregulated, regulating proliferation, apoptosis evasion, and invasive behavior [64]. Conversely, miR-151a is downregulated in exosomes, suggesting potential tumor-suppressive functions. This pattern of upregulated oncogenic miRNAs combined with downregulated suppressor miRNAs illustrates the complexity of exosomal RNA cargo and its role in fine-tuning tumor progression.

In melanoma, serum-derived exosomes enriched with miR-542–5p and miR-106b have been reported as potent circulating biomarkers [65]. These miRNAs contribute to cell cycle regulation and immune escape, reflecting the aggressive biology of advanced melanoma. Similarly, in bladder cancer, urinary exosomes carrying miR-375 and miR-146a are significantly elevated in patients and correlate with tumor burden and recurrence risk [66]. The use of urine as a sample matrix highlights the feasibility of cancer-type–specific liquid biopsy strategies.

Exosomal miRNAs are also crucial in renal cell carcinoma (RCC), where serum-derived miR-210 has been validated as a biomarker [67]. Functionally, miR-210 is induced under hypoxic conditions and regulates mitochondrial metabolism, angiogenesis, and resistance to apoptosis. Its enrichment in RCC exosomes thus provides a mechanistic explanation for its diagnostic relevance.

In hepatocellular carcinoma (HCC), exosomal miR-122 and miR-148a are markedly downregulated [68]. Since miR-122 is normally abundant in hepatocytes and regulates lipid metabolism and proliferation, its reduction reflects malignant transformation and hepatocyte dysfunction. The diagnostic significance of these miRNAs lies in their ability to distinguish HCC patients from healthy individuals and those with chronic liver disease.

Hematologic malignancies also exhibit exosome-associated miRNA alterations. In diffuse large B-cell lymphoma (DLBCL), plasma exosomal miR-155 is consistently elevated [69]. As a well-characterized oncomiR, miR-155 promotes B-cell proliferation and survival by targeting SHIP1 and SOCS1, linking its circulating abundance directly to lymphoma pathogenesis. Finally, in esophageal squamous cell carcinoma (ESCC), the serum exosomal miR-1246/miR-106b ratio has been validated as a diagnostic marker [70]. Combined use of these two miRNAs provides higher discriminatory power than either alone, highlighting the value of ratio-based biomarkers in clinical diagnostics.

In addition to tumor-cell–derived miRNAs, several exosomal miRNAs released from immune, stromal, and endothelial cells have demonstrated diagnostic promise by capturing early alterations in the tumor microenvironment. Immune-derived exosomal miRNAs, such as miR-155 and miR-21, mirror the balance between antitumor inflammation and immunosuppressive signaling, showing elevated levels in circulation even before overt tumor formation [71,72]. Stromal-derived miRNAs, including miR-92a-3p and miR-222, participate in fibroblast-mediated extracellular-matrix remodeling and epithelial–mesenchymal transition, processes that accompany the earliest stages of malignant transformation [73,74]. Endothelial-cell–derived exosomal miRNAs, notably miR-126 angiogenic and hypoxic pathways and are dynamically modulated during pre-vascular tumor development [75]. These cell-specific exosomal miRNAs collectively reflect the crosstalk between tumor and surrounding tissues, serving as sensitive, non-invasive indicators of nascent oncogenic activity. Integrating such non-tumor-cell–derived miRNA signatures with conventional tumor-derived panels could further enhance the sensitivity and specificity of liquid-biopsy-based early cancer detection.

### 2.3 Other exosomal biomarker

In addition to proteins and miRNAs, exosomes carry a wide range of other biomolecules that are increasingly recognized as promising cancer biomarkers. These include long non-coding RNAs (lncRNAs), circular RNAs (circRNAs), PIWI-interacting RNAs (piRNAs), and double-stranded DNA (exo-DNA). Because these molecules are enclosed within the exosomal lipid bilayer, they are shielded from enzymatic degradation, remain highly stable in circulation, and can accurately reflect the genetic and epigenetic landscape of the parental tumor cells.

Exosomal lncRNAs, which are transcripts longer than 200 nt, modulate chromatin architecture and transcriptional programs and are often selectively packaged into exosomes by RNA-binding proteins such as hnRNPA2B1 and YBX1. Several lncRNAs have demonstrated clear diagnostic value. For instance, exosomal H19 is markedly up-regulated in the serum of gastric-cancer patients, decreases after surgical tumor removal, and correlates with TNM stage, indicating its usefulness for non-invasive monitoring [76]. In non-small-cell lung cancer (NSCLC), exosomal TBILA and AGAP2-AS1 are elevated in patient sera, showing positive associations with tumor size and pathological stage [77], while MALAT1 is abundant in NSCLC-derived exosomes and enhances proliferation and migration via the PI3K/Akt pathway [78]. In hepatocellular and breast cancers, other lncRNAs have also been shown to influence angiogenesis, macrophage polarization, and chemoresistance, supporting the concept that exosomal lncRNAs convey mechanistic information on tumor progression.

CircRNAs, generated by back-splicing of exons into covalently closed loops, exhibit exceptional resistance to exonuclease degradation and display cancer-type specificity. Serum exosomal hsa-circ-0004771 is markedly elevated in colorectal-cancer (CRC) patients, including those with early-stage disease, and decreases after surgical resection, suggesting value for early detection and post-operative surveillance [79]. Another CRC-related molecule, exosomal circGAPVD1 (hsa-circ-0003270), is enriched in patient plasma and correlates with lymph-node metastasis and TNM stage [80]. Mechanistically, many circRNAs act as miRNA sponges or scaffolds for RNA-binding proteins, thereby modulating oncogenic signaling in recipient cells.

PIWI-interacting RNAs (piRNAs) represent another emerging class of exosomal small RNAs. Although once considered germline-restricted, recent studies have verified their presence in tumor-derived exosomes. A serum exosomal five-piRNA panel (piR-1029, piR-15254 and others) was reported to distinguish hepatocellular-carcinoma patients from healthy donors with AUROC >0.90, even at early disease stages [81]. These findings suggest that exosomal piRNAs add an additional regulatory and diagnostic layer beyond miRNAs.

Furthermore, tumor-derived exosomes carry double-stranded genomic DNA fragments spanning all chromosomes. This exo-DNA contains driver mutations identical to those found in the parental tumor, providing a complementary analyte to cell-free DNA (cfDNA). In pancreatic ductal adenocarcinoma, exosomal DNA harboring KRAS and TP53 mutations was found to perfectly match tissue genotyping results [82]. Unlike cfDNA, exo-DNA shows higher integrity and tumor specificity because it is physically protected by the exosomal membrane. Altogether, these molecular classes, such as lncRNAs, circRNAs, piRNAs, and exo-DNA, expand the repertoire of exosomal biomarkers and offer additional routes for precise, multiplexed liquid-biopsy diagnostics.

Table 1 provides a comprehensive summary of exosomal biomarkers for cancer diagnosis, consolidating key findings from recent studies.

**Table 1:** Summary of exosomal biomarkers for cancer diagnosis.

Parent cell of the exosome	Type	Marker	Biofluids	Trend	Ref
Breast cancer	Protein	Glypican-1 (GPC1)	Serum exosomes	Upregulation	[43]
Breast cancer	Protein	HER2 (full-length, truncated)	Serum exosomes	Upregulation	[44]
Breast cancer	Protein	CD24, CD82	Serum exosomes	Upregulation	[45]
Breast cancer	miRNA	miR-21	Serum exosomes	Upregulation	[58]
Breast cancer	miRNA	miR-1246	Serum exosomes	Upregulation	[83]
Lung cancer	Protein	EGFR	Serum exosomes	Upregulation	[48]
Lung cancer	miRNA	miR-126, miR-21	Plasma exosomes	Down (miR-126), Up (miR-21)	[59]
Colorectal cancer	Protein	CD147	Serum exosomes	Upregulation	[47]
Colorectal cancer	miRNA	let-7a, miR-1229, miR-1246, miR-150, miR-21, miR-223, and miR-23a	Serum exosomes	Upregulation	[60]
Colorectal cancer	circRNA	hsa-circ-0004771	Serum exosomes	Upregulation	[79]
Colorectal cancer	circRNA	circGAPVD1 (hsa-circ-0003270)	Plasma exosomes	Upregulation	[80]
Pancreatic cancer	Protein	Glypican-1 (GPC1)	Serum exosomes	Upregulation	[84]
Pancreatic/Breast cancer	Protein	Integrins (αvβ5, α6β4)	Serum exosomes	Upregulation	[85]
Pancreatic cancer	miRNA	miR-196a, miR-1246	Serum exosomes	Upregulation	[86]
Pancreatic cancer	DNA	KRAS, TP53 mutations	Plasma exosomes	Upregulation	[82]
Prostate cancer	Protein	PSA (exosomal form)	Urine exosomes	Upregulation	[51]
Prostate cancer	miRNA	miR-141, miR-375	Plasma exosomes	Upregulation	[61]
Ovarian cancer	Protein	EpCAM, CD24	Ascites exosomes	Upregulation	[46]
Ovarian cancer	miRNA	miR-141, miR-200 family (miR-200a, −200b, −200c), miR-203, miR-205, and miR-214.	Serum exosomes	Upregulation	[62]
Ovarian cancer	miRNA	miR-21	Serum exosomes	Upregulation	[87]
Gastric cancer	miRNA	miR-423–5p	Serum exosomes	Upregulation	[63]
Gastric cancer	lncRNA	H19	Serum exosomes	Upregulation	[76]
Glioblastoma	Protein	EGFRvIII	Serum exosomes	Upregulation	[49]
Glioblastoma	miRNA	miR-9 miR-29 b miR-34 b-3p miR-222,	Plasma exosomes	Upregulation	[64]
Glioblastoma	miRNA	miR-151a	CSF, Serum Exosome	Downregulation	[64]
Melanoma	miRNA	miR-542–5p miR-106b	Serum exosomes	Upregulation	[65]
Bladder cancer	miRNA	miR-375, miR-146a	Urine exosomes	Upregulation	[66]
Renal cell carcinoma	miRNA	miR-210	Serum exosomes	Upregulation	[67]
Hepatocellular carcinoma	miRNA	miR-122, miR-148a	Plasma exosomes	Downregulation	[68]
Hepatocellular carcinoma	piRNA	piR-1029, piR-15254	Serum exosomes	Upregulation	[81]
NSCLC	lncRNA	TBILA, AGAP2-AS1	Serum exosomes	Upregulation	[77]
NSCLC	lncRNA	MALAT1	Serum exosomes	Upregulation	[78]
DLBCL (lymphoma)	miRNA	miR-155	Plasma exosomes	Upregulation	[69]
Esophageal squamous cell carcinoma	miRNA	miR-1246	Serum exosomes	Upregulation	[70]
Macrophage	Protein	PD-L1	Plasma exosomes	Upregulation	[51]
Macrophage	miRNA	miR-155	Plasma exosomes	Upregulation	[71]
Macrophage	miRNA	miR-21	Serum exosomes	Upregulation	[72]
Dendritic cells	Protein	MHC II, CD86	Serum exosomes	Upregulation	[52]
CAF	Protein	QSOX1	Serum exosomes	Upregulation	[53]
CAF	Protein	Tenascin-C, periostin	Serum exosomes	Upregulation	[54]
CAF	miRNA	miR-92a-3p	Serum exosomes	Upregulation	[73]
CAF	miRNA	miR-222	Serum exosomes	Upregulation	[74]
Endothelial cell	Protein	DEL-1	Plasma exosomes	Upregulation	[55]
Endothelial cell	Protein	VEGF, Angiopoietin-2	Plasma Exosomes	Upregulation	[56]
Endothelial cell	miRNA	miR-126	Plasma Exosomes	Downregulation	[75]

## 3 Application of nanomaterials in exosome isolation

### 3.1 Nanoparticle-based exosome isolation by centrifugation

A major bottleneck in exosome research is the lack of rapid, efficient, and accessible isolation methods. Conventional differential ultracentrifugation, though widely used, requires specialized equipment, extremely high centrifugal forces (>100,000×*g*), and long processing times, which limit throughput and hinder clinical translation. To overcome these challenges, nanoparticle-based immuno-weighting strategies have been developed, in which exosomes are selectively captured by functional nanostructures that increase their effective density and mass, allowing precipitation at much lower centrifugal forces. This approach has gained considerable attention because it not only streamlines sample preparation but also enhances selectivity for specific exosome subpopulations.

A representative example of this concept is the AuNP-based small-exosome isolation method described by Guru et al. [88]. In this study, polyethylene glycol (PEG)-stabilized AuNPs were covalently conjugated with anti-CD63 antibodies via EDC/SNHS chemistry, enabling specific binding to CD63-positive vesicles. When incubated with serum samples, the AuNP-exosome complexes gained sufficient additional mass to sediment under low-speed centrifugation, eliminating the need for ultracentrifugation. This system was particularly effective for isolating small exosomes (<50 nm), which are often underrepresented in conventional workflows due to their lower buoyant density. The method was validated using dynamic light scattering (DLS), nanoparticle tracking analysis (NTA), and transmission electron microscopy (TEM), as well as western blotting of canonical exosomal proteins. Achieving rapid, size-selective isolation within approximately 2 h using bench-top centrifugation highlights the feasibility of immuno-weighted precipitation for clinical workflows. Importantly, PEGylation minimized nanoparticle aggregation and non-specific adsorption of serum proteins, underscoring the critical role of surface chemistry optimization.

**Fig. 3:**
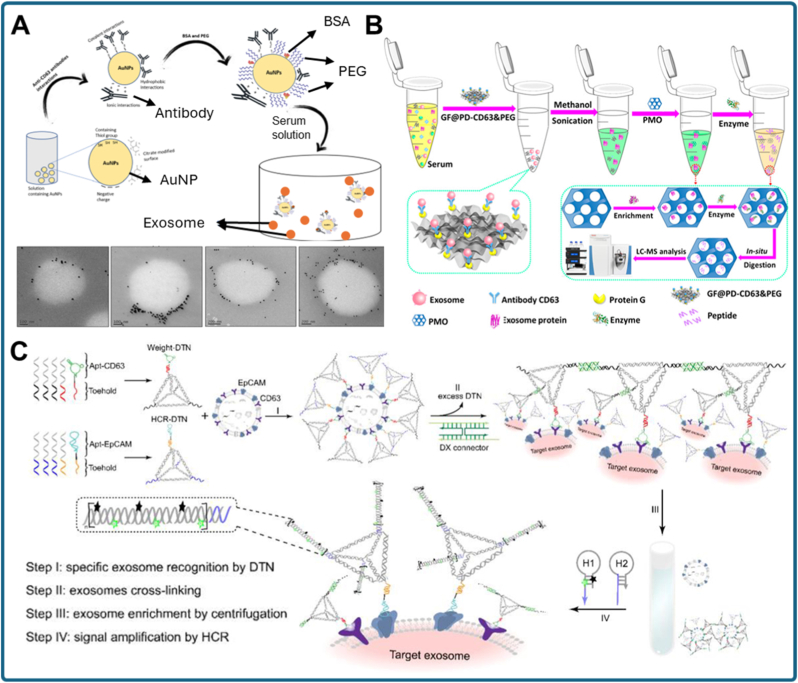
Nanoparticle-enabled centrifugation strategies for exosome enrichment. (a) AuNPs conjugated with anti-CD63 IgY antibodies capture serum exosomes, and the increased particle weight enables sedimentation under low-speed centrifugation [89]. (b) Integrated graphene foam (GF)/periodic mesoporous organosilica (PMO) platform: anti-CD63-modified GF captures exosomes, followed by PMO-assisted protein extraction and digestion to enhance membrane proteomic profiling [90]. (c) DNA-nanoweight-assisted centrifugation: aptamer-decorated DNA tetrahedra cross-link target exosomes, facilitating rapid precipitation and enabling highly sensitive quantification through hybridization chain reaction (HCR) amplification [91].

Building upon the immuno-weighting concept, an alternative strategy utilized IgY antibodies immobilized on AuNPs [89]. Unlike mammalian IgG antibodies, which are more costly to produce and purify, IgY antibodies derived from chicken eggs offer a scalable and economical source. In this approach, IgY antibodies specific to exosomal tetraspanins (CD9, CD63, CD81) were physically adsorbed onto PEG-stabilized AuNP surfaces (Fig. 3A). Following incubation with serum, a simple bench-top centrifugation step was sufficient to pellet the exosome–AuNP complexes. This strategy reduced both the cost and complexity of reagent preparation while maintaining specificity for exosomal subpopulations, demonstrating how low-cost antibody sources combined with nanoparticle weighting may facilitate practical large-scale or POC applications.

While the above studies emphasize exosome isolation, subsequent molecular analysis is often constrained by the difficulty of recovering intact proteins, particularly membrane-associated proteins that are poorly solubilized by standard extraction methods. To address this challenge, Fang et al. developed an integrated nanomaterial-based platform that couples exosome capture with enhanced proteomic analysis (Fig. 3B) [90]. In this system, hydrophilic graphene foam (GF) functionalized with anti-CD63 antibodies served as the capture substrate, selectively binding exosomes from complex biological fluids. The vesicles were then treated with methanol to release membrane proteins, which were subsequently adsorbed and digested on periodic mesoporous organosilica (PMO). Acting as a nano-reactor, the PMO facilitated rapid enzymatic digestion and efficient peptide recovery. Compared to commercial isolation kits and conventional in-solution digestion, this integrated GF/PMO workflow identified a substantially greater number of proteins, including a significantly higher fraction with multiple transmembrane domains, thereby enhancing the depth and quality of exosomal proteome profiling. Although this platform does not rely solely on nanoparticle weighting for sedimentation, it demonstrates how nanomaterial-based designs can extend the centrifugation concept into downstream analytical improvements, providing more comprehensive molecular insights.

In addition to metallic nanoparticles, nucleic acid nanostructures have emerged as versatile alternatives for centrifugation-assisted enrichment. A particularly innovative example is the DNA-nanoweight-assisted centrifugation strategy proposed by Lu et al. (Fig. 3C) [91]. This approach employs two distinct DNA tetrahedron nanostructures (DTNs). The first DTN is decorated with multiple aptamers to cross-link exosomes into higher-order aggregates, significantly enhancing their sedimentation efficiency under low-speed centrifugation. The second DTN simultaneously recognizes target surface markers and initiates a hybridization chain reaction (HCR), enabling ultrasensitive downstream quantification. This dual-DTN system achieves enrichment and detection of cancer-related exosomes at concentrations as low as ∼1.8 × 10^2^ vesicles per μL in clinical serum, representing up to a 1000-fold improvement in sensitivity over conventional ELISA assays. Importantly, the modularity of DNA nanostructures allows rapid redesign for different biomarkers by simply exchanging aptamer sequences, making this approach a flexible and powerful platform for POC diagnostics.

Collectively, these studies highlight the diversity of nanoparticle-enabled centrifugation methods for exosome isolation, while also revealing remaining challenges. AuNP-based immuno-weighting is inherently limited by antibody specificity, potentially biasing enrichment toward tetraspanin-rich vesicles and excluding heterogeneous exosome populations. Non-specific protein adsorption and aggregation, although mitigated by PEGylation, can still compromise purity. DNA nanostructure-based weighting raises concerns regarding stability in biological fluids, potential immunogenicity, and the complexity of oligonucleotide synthesis. Furthermore, although centrifugation forces are reduced, these methods still rely on benchtop centrifuges, which may limit deployment in resource-constrained settings without additional engineering adaptations. Despite these limitations, the potential for rapid, selective, and low-equipment exosome isolation is clear. By integrating immuno-weighting, programmable nanostructures, and downstream analytical enhancements, nanoparticle-based centrifugation methods are moving closer to clinical translation. Future directions may focus on multiplexed systems capable of isolating and characterizing exosomes based on multiple biomarkers simultaneously, integrating isolation with amplification-free detection schemes, and adapting these platforms into fully automated microfluidic devices. Overall, nanoparticle-enabled centrifugation represents a crucial step toward bridging laboratory-scale exosome research with practical, real-world diagnostic applications.

### 3.2 MNP-based exosome isolation by magnetic force

MNPs have become powerful tools for exosome isolation due to their large surface-to-volume ratios, tunable surface chemistry, and responsiveness to external magnetic fields. Unlike ultracentrifugation, which requires prolonged spin times and specialized equipment, magnetic isolation enables rapid, selective, and minimally invasive recovery of exosomes under low shear stress. By conjugating MNPs with affinity ligands such as antibodies, aptamers, or host–guest chemistries, researchers have achieved highly efficient capture of tumor-derived vesicles from complex biological matrices. Many designs further integrate magnetic enrichment with downstream detection, paving the way for POC diagnostic applications.

**Fig. 4:**
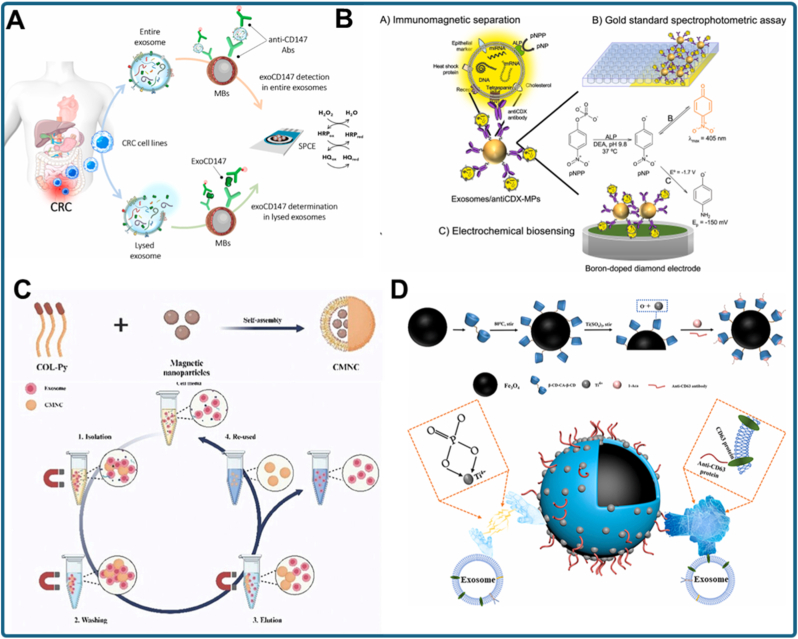
MNP-based exosome isolation strategies. (a) Immunomagnetic capture of CD147-positive exosomes combined with electrochemical biosensing for early colorectal cancer detection [92]. (b) Magnetic enrichment of ALP-positive breast cancer exosomes, using intrinsic alkaline phosphatase activity as a direct reporter [93]. (c) Chitosan oligosaccharide lactate/pyrene-based self-assembled magnetic nanoclusters for efficient exosome separation [94]. (d) Bifunctional immunoaffinity MNPs integrating host–guest interactions with antibody targeting for high-efficiency exosome capture [95].

An early demonstration of the diagnostic potential of immunomagnetic exosome capture targeted CD147-positive vesicles in colorectal cancer (Fig. 4A) [92]. Anti-CD147-modified MNPs selectively enriched TDEs, which were subsequently analyzed via electrochemical biosensing. This integrated workflow reduced background interference and enabled early detection of colorectal cancer signatures directly from serum, illustrating how magnetic force can facilitate both enrichment and high-sensitivity biosensing in a single platform. Similarly, the intrinsic enzymatic activity of exosomal alkaline phosphatase (ALP) has been exploited to simplify biosensor design (Fig. 4B) [93]. In this system, ALP-positive exosomes were isolated by immunomagnetic beads, and their endogenous enzymatic activity served as a direct reporter, eliminating the need for synthetic labeling. This approach demonstrates that magnetic isolation can enrich specific subpopulations while leveraging natural exosomal functions for detection.

In addition to immunocapture, self-assembled nanoclusters have been employed to improve recovery efficiency. For example, chitosan oligosaccharide lactate and 1-pyrenecarboxylic acid were used to form MNP-based nanoclusters with high surface reactivity (Fig. 4C) [94]. These clusters provided multiple binding sites and strong magnetic responsiveness, enabling rapid separation of exosomes while preserving vesicle integrity. In another strategy, bifunctional immunoaffinity MNPs were engineered using a host–guest interaction system, combining cyclodextrin-based recognition with antibody targeting (Fig. 4D) [95]. This dual-affinity design allowed selective binding and efficient magnetic separation, enhancing recovery compared to single-ligand systems. Even minimalistic approaches, such as Fe_2_O_3_ nanoparticles, have proven sufficient for cost-effective exosome enrichment, highlighting the versatility of magnetic designs for downstream analysis [96]. Recent advances extend beyond enrichment to enable direct detection. For instance, Au-loaded nanoporous ferric oxide nanozymes simultaneously isolate and analyze exosomes [97]. By exploiting the peroxidase-like activity of the Au–Fe_2_O_3_ composite, this system eliminates the need for pre-isolation steps, combining magnetic capture and colorimetric signal generation in a single workflow. This multifunctional approach illustrates how magnetic nanomaterials can serve both as separation agents and biosensing catalysts, streamlining exosome analysis. Similarly, magnetic separation has been successfully integrated into microfluidic.

Platforms, as demonstrated with a nickel-doped microfluidic chip [98]. Embedding Ni-doped structures within the chip allows rapid, efficient immunomagnetic capture of breast cancer-derived exosomes, coupled with on-chip detection. This design reduces assay time, minimizes sample consumption, and demonstrates the potential of immunomagnetic microfluidic systems for PO testing.

Collectively, these studies underscore the versatility of MNP-based strategies for exosome isolation. Nevertheless, several challenges persist. Antibody-functionalized MNPs can be costly and may preferentially enrich tetraspanin-positive subpopulations, introducing bias into exosome profiling. Chitosan- or host–guest-based systems broaden binding specificity but may be affected by batch-to-batch variability. Nanozyme-enabled approaches simplify workflows but require careful optimization to minimize non-specific catalysis. Likewise, although microfluidic integration enhances automation and shortens assay time, fabrication costs and the lack of standardization still hinder large-scale implementation. Despite these limitations, magnetic force-driven isolation provides a rapid, selective, and potentially multifunctional alternative to ultracentrifugation for exosome research and clinical diagnostics. The field is steadily progressing from simple capture toward integrated nanoplatforms that unify isolation, biomarker detection, and multiplexing within a single device. With continued advances in surface chemistry, reproducibility, and clinical validation, MNP-based systems are poised to play a pivotal role in translating exosome biomarkers into practical liquid biopsy applications.

### 3.3 Nanoporous structure-based separation by size difference

Size-based separation is one of the most fundamental label-free approaches for exosome isolation, exploiting their nanoscale dimensions (30–150 nm) relative to proteins, lipoproteins, and cellular debris. Nanoporous membranes and microfluidic devices engineered with controlled pore sizes or trapping geometries enable selective, scalable, and reproducible enrichment of vesicles. Recent advances in nanostructured membranes, tangential flow filtration (TFF), and hydrodynamic trapping chips have markedly improved recovery yield and purity while facilitating integration with downstream biosensing.

**Fig. 5:**
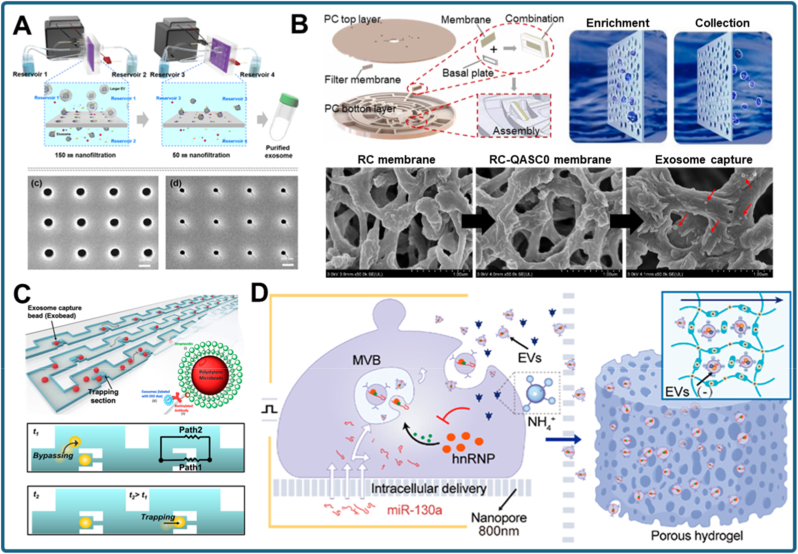
Nanoporous structure-based strategies for size-selective exosome separation. (a) Electrophoretic oscillation–assisted tangential flow filtration (EO–TFF) with antifouling membrane modification for efficient isolation of bovine milk exosomes [99]. (b) Microfluidic chip integrating nanoporous membrane separation with aptamer-based recognition for sensitive detection of exosomal membrane proteins in lung cancer diagnosis [100]. (c) Hydrodynamic trapping microfluidic device retaining exosomes via size-selective flow dynamics while removing smaller proteins and larger debris [101]. (d) Scalable and facile size-based exosome purification and analysis using a hydrodynamic trapping microfluidic device [102].

A representative example of nanoporous membrane technology is the electrophoretic oscillation–assisted TFF (EO–TFF) system developed for bovine milk exosome isolation. In this approach, micro- and ultrafiltration membranes were modified with antifouling coatings to prevent protein deposition and filter clogging (Fig. 5A) [99]. Electrophoretic oscillation was applied to mitigate concentration polarization, thereby enhancing filtration efficiency and recovery yield. This study illustrates how classical filtration can be augmented with external fields to improve selectivity. Building on this principle, recent work has miniaturized TFF into microfluidic devices for plasma-derived exosomes. [103]. A double-TFF configuration significantly improved enrichment efficiency and reduced fouling compared to single-stage setups, while another microfluidic TFF device achieved high-purity exosome purification directly from human plasma [104]. Together, these advances show how TFF systems have evolved from bulk membrane filtration to integrated, high-performance microfluidic platforms suited to clinical workflows.

In parallel, nanoporous structures have been combined with molecular recognition modules to enable simultaneous isolation and detection. Zhao et al. developed a microfluidic device incorporating nanoporous membranes for exosome enrichment, followed by aptamer-based recognition of membrane proteins for lung cancer diagnostics (Fig. 5B) [100]. This design provided not only size-selective separation but also high sensitivity in downstream biomarker analysis, minimizing sample requirements. A complementary platform integrated nanoporous isolation with a chemiluminescence biosensor [105]. By coupling HCR amplification with size-based preconcentration, it enabled multiplexed and ultrasensitive detection of exosomal miRNAs. Both studies underscore the trend toward functionalized nanoporous chips that merge physical filtration with nucleic acid or protein biosensing, streamlining workflows for POC testing.

A third class of devices leverages hydrodynamic trapping and nanostructured microenvironments. Tayebi et al. introduced a simple microfluidic hydrodynamic trapping device in which exosomes are retained within channel constrictions based on flow dynamics, while smaller proteins and larger debris are flushed away (Fig. 5C) [101]. This purely physical strategy avoids reliance on surface markers, ensuring unbiased vesicle recovery. Building on this concept, Wang et al. created an integrated nanoporous platform (PURE) that combines nanopore electroporation, ammonium removal, and porous hydrogel-based in situ exosome capture (Fig. 5D) [102]. This system achieved an in situ capture efficiency of 87.1 % and produced engineered EVs with yields about 12-fold higher than natural secretion, while enabling therapeutic cargo loading up to 146-fold enrichment.

In a related study, Zhao et al. used microsphere-mediated isolation integrated with dielectrophoresis to physically constrain and electrically manipulate exosomes, increasing trapping efficiency and allowing downstream ultrasensitive detection [106]. Extending this paradigm, Li et al. developed a 3D self-assembled nanostructured SiO_2_ microfluidic chip [107]. Its three-dimensional nanoporous matrix provided a large surface area and interconnected pore network that greatly enhanced exosome capture and recovery, enabling highly sensitive analyses. These examples highlight the versatility of microfluidic hydrodynamic and nanostructured platforms in achieving efficient, label-free separation through combined flow engineering and nanoscale porosity.

In summary, nanoporous structure-based exosome isolation offers several notable advantages: it is label-free, compatible with continuous-flow operation, and readily adaptable to integration with downstream assays. TFF-based devices (EO–TFF, double TFF, plasma purification) emphasize scalability and antifouling properties, making them attractive for clinical translation. Hybrid microfluidic chips (aptamer- and chemiluminescence-integrated) illustrate analytical multifunctionality, whereas hydrodynamic and nanostructured devices achieve high physical precision and sensitivity without relying on biochemical labels. Despite these advances, several key challenges persist. Membrane fouling, vesicle deformation under high transmembrane pressures, and limited recovery of specific subpopulations persist as practical obstacles. Future efforts are expected to focus on smart antifouling coatings, adaptive pore architectures, and integration with multiplexed biosensing, ultimately advancing nanoporous separation into a robust and clinically viable exosome isolation strategy.

### 3.4 Comparative analysis and technological insights of nanomaterials-based exosome isolation

The diverse nanomaterial-assisted strategies described above highlight significant progress toward efficient exosome isolation, yet their relative performance varies widely depending on the physical principles, surface chemistry, and operational context involved. To critically assess their strengths and limitations, several parameters must be considered, including recovery yield, purity, processing time, matrix compatibility, operational complexity, and scalability. Recovery yield represents the fraction of vesicles successfully collected relative to input, while purity reflects the degree of separation from lipoproteins and protein aggregates. Processing time and user intervention determine the feasibility for POC testing, whereas cost and automation potential define clinical scalability. Furthermore, each isolation mechanism introduces specific biases that shape the subpopulation of exosomes ultimately analyzed, which can influence downstream biomarker quantification.

Among the examined materials, magnetic nanoparticles (MNPs) exhibit the highest degree of versatility and POC readiness. Their magnetic responsiveness enables rapid, low-shear enrichment of target vesicles without specialized equipment, facilitating integration into disposable cartridges or automated magnetic racks [108]. Owing to their high surface-to-volume ratios and tunable surface chemistry, MNPs provide efficient immunoaffinity capture of tetraspanin-positive exosomes (CD9, CD63, CD81) and have been validated across diverse biofluids including plasma, urine, and saliva. Their major advantages include simplicity, reproducibility, and minimal sample loss; however, antibody functionalization adds cost, and their specificity can bias toward subpopulations, overlooking exosomes with low marker expression. In addition, potential carry-over of capture ligands and inter-batch surface variability require rigorous quality control. Despite these challenges, MNP-based isolation remains the most clinically adaptable method, particularly for rapid or multiplexed workflows.

Gold nanoparticles (AuNPs) provide complementary advantages based on their dual optical and biochemical functionality [109]. In immuno-weighting strategies, AuNP conjugation increases the effective density of exosome–nanoparticle complexes, allowing precipitation by low-speed centrifugation. This approach eliminates the need for ultracentrifugation and can be performed with standard laboratory equipment. AuNPs also serve as an optical or electrochemical interface for subsequent detection, enabling integration of isolation and signal readout within the same nanostructure. Nevertheless, their high surface energy results in non-specific adsorption of serum proteins, and uncontrolled aggregation can compromise reproducibility. These issues necessitate precise optimization of PEGylation, ionic strength, and ligand density [110]. Although AuNP-based enrichment offers strong analytical performance, it is less suitable for field-deployable POC systems due to its dependence on centrifugation, but it remains valuable for laboratory-scale integrated sensing assays.

DNA nanostructures, including tetrahedral and origami-like architectures, offer a radically different paradigm based on programmable self-assembly and multivalent molecular recognition. These constructs present aptamers in defined three-dimensional geometries, enabling highly specific cross-linking and precipitation of target exosomes under gentle conditions. Their key strength lies in the molecular precision and exceptional selectivity achieved through rational sequence design, which minimizes off-target binding and allows the incorporation of amplification modules such as HCR. Consequently, DNA nanostructures can achieve purities exceeding 90 % and enrich rare vesicle subtypes that conventional immunocapture may miss. However, their synthetic cost, limited stability in biological fluids due to nuclease degradation, and potential immunogenicity hinder widespread clinical translation [111]. These features make them ideal for research or discovery-oriented applications requiring precise molecular profiling rather than routine diagnostics.

In contrast, nanoporous and hybrid materials, including graphene foam, periodic mesoporous PMO, and TFF membranes, provide label-free isolation based on size exclusion and physical trapping. These platforms are well suited for large-volume clinical samples because they enable continuous flow and scalability without expensive ligands. Moreover, nanostructured surfaces can be combined with on-chip proteomic digestion or chemiluminescent detection, transforming the isolation step into an analytical interface. Despite these advantages, nanoporous membranes face persistent challenges such as fouling, concentration polarization, and mechanical deformation of vesicles under high transmembrane pressures [99]. Engineering improvements, including electro-osmotic oscillation and antifouling coatings, have mitigated these issues but not fully eliminated them. Consequently, these systems offer high throughput and reproducibility for centralized laboratories but remain less adaptable for decentralized or portable diagnostics.

Table 2 provides a comprehensive summary of nanomaterial-based exosome isolation methods, classified by nanomaterial type, isolation principle, recovery yield, purity, and processing time. Comparing these four major classes reveals distinct trade-offs. MNP-based systems offer superior automation compatibility and user convenience, making them ideal for rapid POC or integrated cartridge assays. AuNP-based systems combine isolation with optical or electrochemical sensing, bridging the gap between capture and signal transduction but requiring controlled laboratory environments. DNA nanostructure-based systems provide the highest analytical purity and molecular precision, best suited for biomarker discovery or validation of low-abundance exosome subsets. Nanoporous and hybrid membranes deliver unparalleled scalability for large-volume samples and multi-sample processing, supporting clinical laboratories where throughput outweighs single-sample sensitivity. Thus, a single nanomaterial platform is not universally optimal, the choice must match the intended diagnostic context and balance sensitivity, specificity, cost, and throughput.

**Table 2:** Summary of nanomaterial-based exosome isolation methods.

Nanomaterial system	Isolation principle	Yield (%recovery)	Purity	Processing time	Technical complex	Ref
Magnetic nanoparticle (MNPs)	Immunocapture under magnetic field	74–102 %	Medium	<1 h	Low (user-friendly)	[88,[91], [92], [93], [94], [95], [96], [97], [98]]
Gold nanoparticles (AuNPs)	Density enhancement + immunoweighting centrifugation	77–80 %	Medium	1–3 h	Medium (high- centrifugation)	[88,89]
DNA nanostructures (DTNs)	Cross-linking + aptamer-based precipitation	480 %	High	3 h	High (complex synthesis/stability issue)	[93]
Graphene/PMO hybrids	Surface adsorption + in situ proteomic digestion	72 %	High	40 min	Medium (Simple/additional enrichment steps)	[101]
Nanoporous membrane	Size-based filtration	77–87 %	Low	1.5–3 h	High (Microfluidic system)	[[99], [100], [101], [102], [103], [104], [105], [106], [107]]

From a translational perspective, MNP and membrane-based systems currently exhibit the greatest potential for regulatory approval due to their mechanical simplicity, biocompatibility, and reproducibility. DNA and AuNP-based systems, while highly sensitive, still face manufacturing and standardization challenges. Addressing these gaps requires harmonious benchmarking such as yield, particle-to-protein ratio, and unresponsive protein removal efficiency to compare platforms under the same conditions. Moreover, the introduction of multi-epitope or hybrid capture designs may mitigate the intrinsic bias toward certain exosome subtypes. Future research should focus on integrating antifouling nanocoatings, reusable ligand scaffolds, and reference particle standards to ensure batch-to-batch reproducibility.

In summary, nanomaterial-based exosome isolation technologies demonstrate complementary strengths across analytical performance, cost, and scalability. MNP and nanoporous systems appear most mature for clinical adaptation, whereas AuNP and DNA nanostructure approaches offer unique precision advantages for advanced multiplexed or molecular assays. A reasonable combination of these strategies, such as magnetically driven DNA hybrid structures or AuNP functionalized membranes, can overcome the limitations of individual systems. These comparative insights establish a conceptual framework for selecting appropriate nanomaterials according to specific clinical or research needs. The purified exosomes obtained through such optimized isolation processes serve as the foundation for subsequent molecular detection, which is explored in the next section focusing on nanomaterial-assisted signal transduction and amplification strategies.

These comparative insights illustrate that rational integration of nanomaterials into exosome isolation requires balancing performance, cost, and scalability. The resulting purified vesicles form the foundation for downstream biomarker analysis. The next section therefore focuses on how nanomaterials further enhance signal transduction and amplification in exosomal biomarker detection for cancer diagnostics.

Beyond performance metrics, a critical biological consideration in exosome isolation is the intrinsic heterogeneity of extracellular vesicle populations. Exosomes vary in size (30–150 nm), cargo composition, and cellular origin, meaning that each isolation method enriches a distinct subpopulation. Immunoaffinity capture preferentially isolates vesicles expressing specific surface proteins, potentially overlooking subtypes lacking these markers. Conversely, size-based approaches such as filtration or TFF recover a broader but less specific population, often contaminated with protein aggregates or larger microvesicles. These biases can markedly influence downstream quantification of nucleic acid and protein biomarkers, leading to inconsistent diagnostic outcomes across platforms. Therefore, future nanomaterial-assisted systems should combine orthogonal separation principles such as sequential immunocapture and size filtration, or adopt multi-marker capture chemistry to ensure a more representative exosome profile for reliable clinical interpretation.

## 4 Application of nanomaterials in exosomal biomarker detection for cancer diagnosis

The detection of exosomal biomarkers, particularly miRNAs and surface proteins, has garnered considerable interest as a non-invasive approach for early cancer diagnosis. However, the inherently low abundance of TDEs in biological fluids and the minute quantities of their molecular cargo pose major challenges. Conventional detection methods such as qPCR and ELISA often fall short in sensitivity, specificity, and operational simplicity.

To overcome these barriers, nanomaterials have been increasingly incorporated into biosensing platforms due to their unique physicochemical properties and functional versatility. Metallic nanoparticles, carbon-based nanostructures, and hybrid composites provide distinct advantages in biomolecular detection [112]. Their high surface-to-volume ratios allow dense immobilization of capture ligands such as antibodies or aptamers, thereby enhancing target-binding probability. In addition, nanomaterials can act as efficient transducers, amplifiers, or scaffolds to boost signal intensity and minimize nonspecific interactions [113]. The integration of nanomaterials into exosomal biomarker analysis has transformed traditional, multistep assays into more compact, highly sensitive, and clinically adaptable systems. These nanostructures not only enable direct readout of molecular interactions but also introduce innovative strategies for signal amplification and multiplexing. This section outlines how nanomaterials function as signal transducers, amplifiers, and indirect enhancers in exosomal biomarker detection, collectively advancing their clinical applicability in cancer diagnostics.

### 4.1 Signal transduction via functional nanomaterials in exosomal biomarker detection

Nanomaterials play a crucial role in converting weak biomolecular recognition events into robust and quantifiable analytical signals. Their diverse physicochemical properties—including conductivity, plasmonic resonance, photoluminescence, and metamaterial effects—offer versatile transduction modalities for exosomal biomarker detection. By integrating nanomaterials into electrochemical, optical, and microfluidic systems, researchers have achieved significant improvements in sensitivity, dynamic range, and multiplexing capacity.

**Fig. 6:**
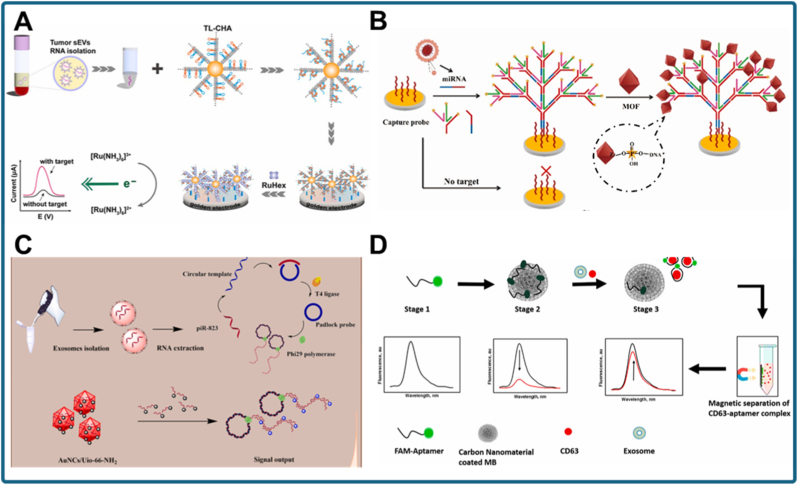
Nanomaterial-enabled signal transduction mechanisms in exosomal biomarker detection. (a) Electrochemical biosensor for sEV-miRNA detection utilizing turbo-like localized catalytic hairpin assembly (TL-CHA) on a nanostructured electrode modified with AuNPs [114]. (b) Plasmonic sensing system employing engineered erythrocytes decorated with Zr MOF as biocompatible substrates for highly sensitive detection of NSCLC-derived exosomal miRNAs [115]. (c) Ratiometric fluorescent assay for exosomal piRNA-823 using Au nanoclusters (Au NCs) embedded in a UiO-66-NH2 metal–organic framework, combined with rolling circle amplification for signal enhancement [116]. (d) Lab-on-a-chip platform for colorectal cancer exosome analysis integrating anti-CD63 aptamers with carbon-coated magnetic (CCM) nanobeads to achieve selective capture and fluorescence signal regulation [117].

Electrochemical approaches remain particularly attractive because of their inherent sensitivity and compatibility with miniaturized devices. A representative example is the turbo-like localized catalytic hairpin assembly (TL-CHA) biosensor for sEV-miRNA detection (Fig. 6A) [114]. In this design, AuNPs were used to construct DNA nanowire scaffolds on the electrode surface, creating a high-surface-area, highly conductive interface. This nanostructured architecture facilitated efficient electron transfer and stabilized nucleic acid hybridization events. Upon recognition of the target exosomal miRNA, the TL-CHA cascade was locally initiated, producing rapid and amplified signal generation.

Beyond electrochemical platforms, nanomaterials have also been engineered to manipulate electromagnetic fields for signal readout. In one study, streptavidin-functionalized terahertz (THz) metamaterials were combined with duplex-specific nuclease (DSN)-triggered rolling circle amplification for pancreatic cancer exosomal miRNA detection [118]. The nanostructured THz metamaterial generated sharp resonance shifts that were highly responsive to dielectric changes induced by amplified DNA products. Streptavidin enabled stable probe immobilization, while the THz substrate achieved attomolar sensitivity. This work illustrates how nanophotonic metamaterials extend signal transduction beyond conventional optical or electrochemical regimes.

Plasmonic nanostructures offer another powerful route for amplifying exosomal biomarker signals. Fan et al. introduced biocompatible engineered erythrocytes decorated with Zr metal-organic framework (MOF) as plasmonic sensor initiators for detecting non-small cell lung cancer (NSCLC)-derived exosomal miRNAs (Fig. 6B) [115]. The erythrocyte membrane served as a flexible and biocompatible scaffold for AuNP assembly, creating a plasmonic substrate with strong localized surface plasmon resonance (LSPR) effects. Target miRNA binding induced spectral shifts that enabled highly sensitive detection. This biomimetic plasmonic interface minimized nonspecific adsorption and provided a scalable path toward clinical application.

Fluorescent nanomaterials have also been exploited for ratiometric and multiplexed detection. A notable example is the Au nanocluster (AuNC)/UiO-66-NH_2_ MOF composite for ratiometric fluorescent detection of exosomal piRNA-823 (Fig. 6C) [116]. In this platform, AuNCs embedded within the MOF acted as fluorescence donors, while the porous MOF scaffold facilitated energy transfer and rolling circle amplification of the target sequence. The resulting changes in emission ratios enabled self-calibrated detection, reducing environmental interference and improving quantitative reliability. This hybrid nanomaterial design demonstrates how MOF-supported nanoclusters can provide sensitive and internally referenced fluorescence signals.

Chinnappan et al. further demonstrated the critical role of nanomaterials in lab-on-a-chip exosome diagnostics (Fig. 6D) [117]. In their study, they developed an “apta-magnetic biosensor” platform by functionalizing magnetic nanobeads with anti-CD63 aptamers. These nanobeads were coated with carbon nanomaterials (graphene), which acted as fluorescence quenchers for the fluorophore-labeled aptamers in the absence of exosomes (OFF state). Upon binding to exosomes, the aptamer detached from the graphene surface, restoring fluorescence (ON state). This combination of magnetic separation and carbon-based signal regulation enabled sensitive, label-free detection of colorectal cancer exosomes, achieving a detection limit of 1457 particles/mL. Overall, this study highlights how functional nanomaterials serve as versatile transducers that convert weak molecular recognition events into robust analytical outputs across electrochemical, optical, and plasmonic modalities.

While single-analyte systems show impressive sensitivity, applying these principles to detect several biomarkers at once brings new challenges. In multiplex exosome detection, it is difficult to keep each signal stable and independent when several recognition reactions occur together. For example, when proteins such as EGFR or EpCAM and nucleic-acid targets like exosomal miRNAs are measured on the same surface, the signals can interfere with one another. Problems such as signal overlap, uneven probe attachment, or competition between binding sites can lead to inaccurate results. These issues become even more complex when optical and electrochemical channels are combined in one device. To overcome them, researchers have developed design strategies such as separating probe areas within microchambers, using color or wavelength-distinct reporters with internal references, and structuring the surface to keep reactions physically apart. These improvements reduce signal interference and make multi-target detection more consistent and reliable.

### 4.2 Signal amplification using nanostructured platforms in exosomal biomarker detection for cancer diagnosis

Nanostructured materials are powerful tools for amplifying detection signals, enabling ultrasensitive analysis of exosomal biomarkers that are often present at very low concentrations in clinical samples. By engineering substrates with nanoscale features such as plasmonic films, metasurfaces, and nanogaps, researchers have exploited strong electromagnetic field confinement, enhanced surface plasmon resonance (SPR), and Raman signal amplification to detect exosomes with unprecedented sensitivity. These platforms not only enhance weak molecular recognition events but also enable label-free and rapid detection, making them highly suitable for cancer diagnostics.

**Fig. 7:**
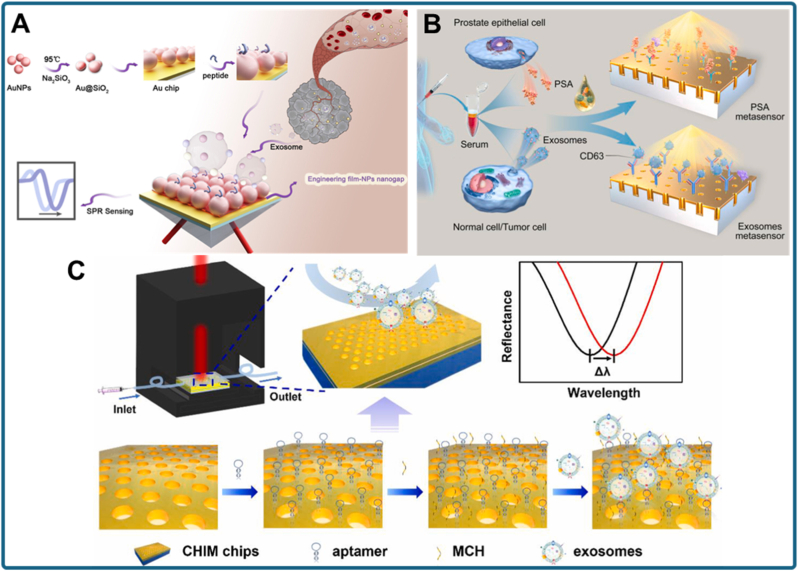
Nanostructured platforms for signal amplification in exosomal biomarker detection. (a) Au@SiO_2_/Au film metasurface engineered as a surface plasmon resonance enhancer for direct and ultrasensitive exosome detection [119]. (b) Label-free plasmonic metasensor for rapid and high-sensitivity detection of prostate-specific antigen (PSA) and exosomes in serum, enabling early prostate cancer diagnosis [120]. (c) Cavity-induced mode hybridization plasmonic sensor providing amplified resonance signals for portable and sensitive exosome detection [121].

A representative example is the development of a tunable Au@SiO_2_/Au film metasurface for direct exosome detection (Fig. 7A) [119]. This design combines an Au–silica core–shell nanoparticle layer with an underlying Au film, creating strong LSPR coupling. The resulting plasmonic hotspots enhance surface sensitivity and allow the detection of intact exosomes without labeling. By tuning the Au@SiO_2_ shell thickness and nanoparticle–film spacing, the metasurface achieves optimized resonance conditions, yielding ultrasensitive readouts with substantial signal amplification. This study demonstrates how engineered nanostructured plasmonic films can convert weak vesicle binding events into strong optical signals.

Following a similar principle, label-free plasmonic metasensing has been applied to simultaneous detection of exosomes and prostate-specific antigen (PSA) in serum (Fig. 7B) [120]. This platform utilizes a gold nanostructured metasurface capable of supporting multiple plasmonic resonances, thereby increasing surface sensitivity. When applied to clinical samples, the metasensor enabled rapid and concurrent detection of PSA and exosomes, facilitating early prostate cancer diagnosis. Notably, the system provided a label-free readout, reducing assay complexity and minimizing processing time. This work illustrates the potential of integrating nanostructured metasurfaces into clinical workflows for rapid, multiplexed, and ultrasensitive exosome-based diagnostics.

Another promising approach involves cavity-induced mode hybridization plasmonic sensors for exosome detection (Fig. 7C) [121]. In this system, nanostructured cavities are engineered to hybridize multiple plasmonic modes, generating sharp resonance peaks and amplified sensitivity. This design allows portable exosome detection by combining strong signal amplification with compact, relatively simple instrumentation. Coupling resonant cavity structures with plasmonic nanomaterials provides both high sensitivity and portability, addressing a major barrier for POC cancer diagnostics.

Surface-enhanced Raman spectroscopy (SERS) represents another powerful amplification strategy, exploiting nanoscale field enhancement in plasmonic nanogaps. A recent study integrated microfluidic devices with SERS-active substrates for exosome biomarker profiling and osteosarcoma diagnosis [122]. Exosomes were enriched within the microfluidic channels and directly interrogated by SERS, producing highly specific molecular fingerprints. The nanostructured plasmonic surfaces amplified the inherently weak Raman signals of exosomal biomolecules, enabling accurate cancer detection from minimal sample volumes. Integrating microfluidics reduced sample handling, enhanced reproducibility, and increased throughput, making this approach highly suitable for clinical translation. Overall, these advances underscore the exceptional analytical sensitivity and diagnostic versatility of nanostructured SERS-based amplification systems.

However, applying this high sensitivity of SERS to detect several biomarkers at once is still challenging. Although SERS can, in theory, distinguish multiple molecules by their unique spectral “fingerprints,” it is difficult to obtain stable and consistent signals when many targets are measured together [123]. The main problems are that the nanoscale “hotspots” where signals are amplified are not evenly formed, different molecules stick to the surface in slightly different ways, and the spectra of each molecule can overlap. To overcome these issues, researchers are developing more uniform nanostructures that generate stable enhancement regions and are also using computer algorithms to separate overlapping spectral patterns. These improvements will make SERS-based methods more reliable and accurate for detecting several exosomal biomarkers in a single test.

### 4.3 Indirect functional roles of nanomaterials in exosomal biomarker detection

While much attention has focused on the direct transducing and amplifying capabilities of nanomaterials in exosomal biomarker detection, their indirect contributions are equally essential for achieving robust and clinically meaningful assays. These roles include providing structural support for probe immobilization, creating antifouling and biocompatible assay environments, enabling pre-concentration and controlled fluid dynamics, and stabilizing reporter molecules. Although these functions do not directly generate detection signals, they establish the foundation for high sensitivity, specificity, and reproducibility.

Graphene-based nanostructures are a prominent example of indirect utility. Graphene quantum dots (GQDs) integrated into field-effect transistor (FET) sensors improve the density and stability of capture probes on the electrode surface. By enhancing probe orientation and electron transfer, GQDs facilitate more efficient hybridization with target exosomal miRNAs, indirectly boosting assay sensitivity even though the nanomaterial itself does not produce a measurable signal [124]. Similarly, hybrid nanocomposites combining AuNPs, GQDs, and graphene oxide (GO) have been used as multiplexed probe interfaces, providing reproducible immobilization surfaces that enhance assay robustness [125]. In these cases, the indirect role of carbon-based nanomaterials lies in creating a high-capacity, stable, and conductive platform that maximizes the performance of biological recognition elements.

MOFs provide another compelling example. Zr-based MOFs with large surface areas and tunable porosity have been employed to stabilize electroactive molecules such as methylene blue, anchoring them near captured exosomes [126]. In this setting, the MOF itself is not the source of the electrochemical signal but serves as a scaffold that improves reporter loading, retention, and reproducibility. This “relay” function ensures stronger and more consistent readouts, illustrating how nanomaterials can indirectly enhance assay quality by controlling the microenvironment around probes and reporters.

Nanostructured TiO_2_ coatings and composites perform similar indirect roles. For instance, Fe_3_O_4_@TiO_2_ beads exploit the strong affinity between TiO_2_ and phosphate groups to capture exosomal lipid bilayers. The magnetic Fe_3_O_4_ core provides manipulability, while the TiO_2_ shell enhances capture stability and minimizes nonspecific adsorption. Detection is performed via SERS immunoassays, but the TiO_2_ primarily acts as a scaffold to facilitate efficient capture and uniform probe presentation [127]. Without such a stabilizing interface, downstream detection would suffer from variability and reduced reproducibility.

In addition to immobilization and scaffolding, nanomaterials also indirectly improve assay specificity by reducing nonspecific binding and background signals. PEGylated surfaces, zwitterionic polymers, and hydrophilic nanocoatings minimize protein fouling and vesicle aggregation. While these coatings do not amplify signals, their antifouling properties are critical for reducing background noise and enabling reliable analysis in complex clinical samples such as plasma or serum.

Another key indirect role is pre-concentration and controlled fluid handling. Porous nanostructures, including silica beads and nanopatterned films, create microenvironments that concentrate exosomes and guide uniform sample flow in microfluidic assays. For example, nanoporous silica scaffolds have been integrated into microchips to trap and concentrate PD-L1-positive exosomes prior to molecular analysis, allowing sensitive detection with minimal sample input [128]. Although these nanostructures do not generate optical or electrochemical signals, their ability to locally enrich analytes greatly enhances detection sensitivity.

Collectively, these examples demonstrate that nanomaterials are not only powerful transducers and signal amplifiers but also indispensable indirect enablers of exosome assays. By providing immobilization scaffolds, stabilizing reporters, reducing nonspecific interactions, and enhancing local analyte concentrations, nanomaterials improve the reproducibility, robustness, and reliability of TAS platforms. As research progresses, the design of multifunctional nanomaterials that simultaneously fulfill both direct and indirect roles will become increasingly important.

### 4.4 Comparative insights into nanomaterial-enabled detection strategies

The preceding subsections have illustrated diverse nanomaterial platforms applied to exosomal biomarker detection. However, despite impressive functional innovations, the analytical performance of each nanomaterial system is governed by distinct physical principles, signal transduction mechanisms, and integration capabilities. A critical comparison reveals the trade-offs among these approaches in terms of sensitivity, selectivity, stability, and clinical adaptability.

Gold-based nanostructures (AuNPs, Au nanorods, Au nanostars) dominate optical and electrochemical sensing due to their strong localized surface plasmon resonance and highly tunable surface chemistry [129]. Their strengths lie in rapid signal generation, broad wavelength tunability, and facile conjugation with thiol- or amine-terminated biomolecules. AuNPs support colorimetric and SERS detection with excellent analytical sensitivity (often femtomolar levels), but signal reproducibility remains a challenge because aggregation, salt concentration, and laser-induced heating can alter plasmonic coupling. Moreover, optical detection is often semi-quantitative and affected by variations in the biological matrix, limiting clinical standardization. Nevertheless, the simplicity of AuNP-based colorimetric assays makes them attractive for low-cost and POC screening.

Magnetic nanoparticles (MNPs), on the other hand, primarily function as dual capture-and-transduction agents in electrochemical or luminescent biosensors. The ability to physically separate exosome-bound probes from complex samples significantly improves signal-to-noise ratios and allows sequential washing and amplification steps within automated cartridges. MNP-based systems achieve high reproducibility and scalability but depend on antibody or aptamer conjugation, which raises cost and introduces capture bias toward specific exosome subtypes [130]. Their signal amplification is generally moderate compared with plasmonic or catalytic systems; however, their robustness and automation readiness render them promising for integrated cartridge-type TAS and regulatory translation.

Carbon-based nanomaterials such as graphene, carbon nanotubes (CNTs), and carbon quantum dots (CQDs) provide unique electronic and photonic advantages. Their π-conjugated networks enable efficient electron transfer and fluorescence quenching, facilitating label-free detection of nucleic acids or surface proteins. Carbon platforms achieve ultra-low background signals and high dynamic ranges, especially in FRET-based miRNA detection. Yet, variability in surface oxidation and heterogeneity between production batches complicate signal normalization [131]. Non-specific π–π adsorption may also suppress weak signals in multiplexed assays. Nonetheless, their low cost, chemical stability, and compatibility with flexible substrates position carbon materials as strong candidates for portable, miniaturized sensing modules.

Metal–organic frameworks (MOFs) and other porous nanomaterials provide an emerging route for high-density probe loading and in situ catalytic amplification. Their large surface area and adjustable pore chemistry allow simultaneous binding of multiple biomarkers or co-encapsulation of enzymes and fluorophores. Although MOFs can achieve extremely high sensitivity through catalytic enhancement, their hydrolytic instability and potential metal-ion leakage limit operation in biological fluids [132]. Moreover, the synthesis and surface modification processes are more complex than those of metallic nanoparticles, which may hinder reproducibility and clinical scale-up.

Hybrid nanocomposites, such as AuNP/graphene, MNP/MOF, or carbon–metal heterostructures, synergistically integrate complementary functionalities to overcome the inherent limitations of each constituent material. By coupling plasmonic enhancement with magnetic separation or conductive transduction, hybrids frequently achieve the best detection limits and multiplexing capability. However, these systems introduce additional fabrication complexity and cost, and batch-to-batch compositional variation can lead to inconsistent analytical performance. Therefore, standardized synthesis and robust interfacial bonding strategies are crucial for reliable translation into routine diagnostics.

To facilitate comparison, Table 3 summarizes representative nanomaterials discussed in Section 4 that have been utilized for exosomal biomarker detection, categorizing them by their composition, sensing mechanism, and analytical role.

**Table 3:** Summary of nanomaterials applied in exosomal biomarker detection. Materials are categorized by structural class and sensing mechanisms, highlighting their physicochemical functions, analytical roles.

Nanomaterial	Representative System	Detection Mechanism/Function	Analytical Role	Ref
**Au-based nanostructures**	AuNPs, Au nanoclusters, Au@SiO_2_/Au film metasurfaces	Localized surface plasmon resonance (LSPR), colorimetric and electrochemical signal enhancement, SERS amplification	Signal transduction and amplification for exosomal proteins and miRNAs	[114,116], [119, 120]
**Magnetic nanoparticles**	Fe_3_O_4_ MNPs functionalized with antibodies or aptamers	Electrochemical and fluorescent readout after magnetic capture/separation	Dual function capture + signal readout	[117]
**Carbon-based nanomaterials**	Graphene, graphene oxide, carbon nanotubes, graphene quantum dots	π–π fluorescence quenching/FRET/electrochemical conduction enhancement	Signal transduction/background suppression in FRET and electrochemical biosensors	[117], [124, 125]
**Metal–organic frameworks (MOFs)**	Zr-MOF, UiO-66-NH_2_ and Zr-MOF modified electrodes	Porous scaffold for fluorophore or enzyme immobilization; ratiometric and catalytic signal amplification	Signal amplification/probe stabilization for miRNA detection	[115,116,126,128]
**Plasmonic/Metamaterial nanostructures**	Terahertz (THz) metamaterials, cavity-mode hybrid plasmonic chips	Resonance shift based on dielectric change during miRNA binding	Label-free resonance sensing for exosomal nucleic acids	[118,121]
**Hybrid nanocomposites**	Fe_3_O_4_@TiO_2_ beads	SERS amplification	Integrated capture + signal amplification/multiplex detection	[127]
**Nanostructured SERS substrates**	Plasmonic films and nanogap arrays	Surface-enhanced Raman fingerprinting of exosomal components	Label-free molecular profiling	[122]

When compared across these categories, Au- and carbon-based nanomaterials generally exhibit the highest analytical sensitivity, whereas MNP- and MOF-based systems provide superior operational robustness, automation compatibility, and scalability. Carbon and hybrid materials support multiplexed or ratiometric detection, whereas MNPs are better suited for POC integration where simplicity and reliability are prioritized over maximal sensitivity. Importantly, exosome heterogeneity and isolation bias must also be considered, as each nanomaterial system interacts differently with vesicle subpopulations.

In summary, the convergence of functional nanomaterials with exosome-specific recognition chemistries has dramatically improved analytical performance. But standardized benchmarking and comparative validation across material classes are still needed. The rational integration of plasmonic nanostructures with magnetic and carbon frameworks therefore represents a promising path toward clinically deployable total analysis systems.

## 5 Nanomaterial-based TAS for exosomal biomarkers

Recently, nanomaterial-based TAS have been introduced as integrated analytical platforms for exosomal biomarker analysis, combining sample preparation, molecular amplification, and signal detection within a single streamlined process. By exploiting the unique physicochemical properties of nanomaterials, these systems enable efficient, sensitive, and potentially on-site diagnostics. Nanoparticles play pivotal roles at multiple stages of TAS, facilitating selective exosome capture, accelerating on-chip biochemical reactions, and amplifying detection signals. Depending on their structural configuration and operational mechanism, nanomaterial-assisted TAS platforms can be broadly categorized into three main types: (1) microfluidic systems that embed nanomaterials within continuous-flow channels, (2) DISK-type systems that employ centrifugal force to automate fluid handling and detection, and (3) vial-type systems that perform the entire analytical workflow in a single reaction tube using multifunctional nanomaterials. The following subsections provide an overview and comparison of these three TAS architectures, highlighting representative examples and their analytical performance.

### 5.1 Microfluidic type TAS for exosomal biomarker detection

Microfluidic-type TAS offer the advantage of integrating exosome isolation, molecular processing, and detection within a single device, reducing assay time and minimizing analyte loss compared with conventional multistep workflows. The incorporation of nanotechnologies—such as MNPs, nanostructured filters, carbon-based nanomaterials, and hybrid nanocomposites—has been instrumental in enhancing capture efficiency, sensitivity, and assay reproducibility. Recent studies demonstrate that these platforms are not only technically robust but also capable of reliably detecting cancer-specific exosomes in patient-derived samples.

**Fig. 8:**
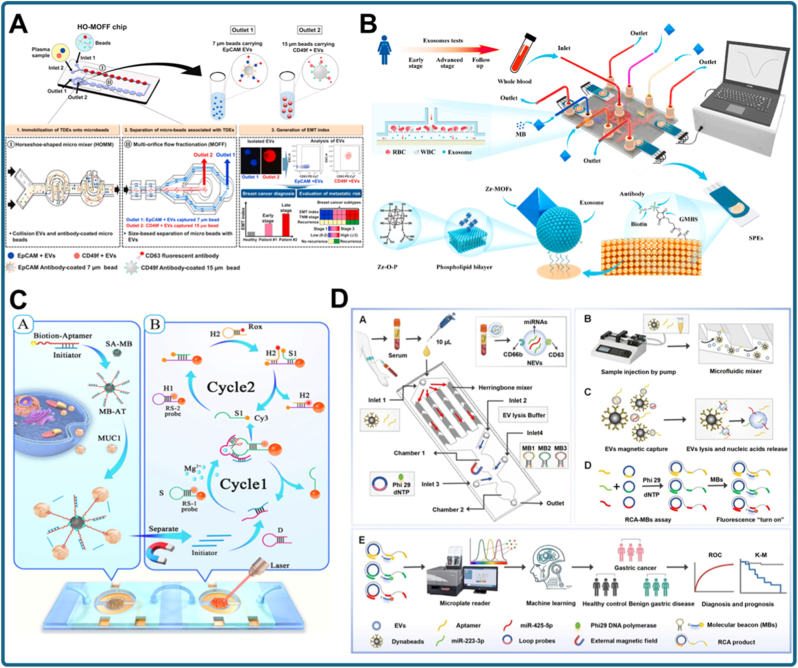
Representative microfluidic-type TAS for exosomal biomarker detection. (a) Microfluidic chip for breast cancer diagnosis and metastatic risk assessment, employing antibody-functionalized nanostructured microchannels to selectively isolate EpCAM/CD49f-positive exosomes from plasma with high sensitivity [133]. (b) Filter–electrochemical microfluidic platform for breast cancer classification, integrating nanoporous filtration membranes with nanostructured electrodes to enable multiplex protein analysis (PMSA, EGFR, CD81, and CEA) [134]. (c) Dual-control microfluidic device combining antibody-coated magnetic nanobead capture, electrokinetic focusing, and dual hybridization chain reaction (HCR) amplification for ultrasensitive detection of tumor-derived exosomal miRNAs in serum [135]. (d) Integrated gastric cancer chip utilizing CD66b antibody-coated nanostructured chambers and on-chip rolling circle amplification (RCA) for high-accuracy analysis of neutrophil-derived exosomal miRNAs (miR-223–3p and miR-425–5p) [136].

A microfluidic chip for breast cancer diagnosis demonstrated rapid and selective isolation of EpCAM- and CD44-positive exosomes from plasma (Fig. 8A) [133]. This device integrated antibody-functionalized microbeads with a microfluidic platform to specifically separate two types of cancer-associated exosomes, enabling both isolation and fluorescence-based detection of early-stage breast cancer in a single system. With a fast isolation speed of 6.7 min per 100 μL and fluorescence detection, this nanoparticle-assisted capture and detection strategy showed excellent diagnostic performance.

In lung cancer diagnostics, an integrative urine-based microfluidic system combined immunomagnetic separation with flow focusing to isolate EGFR- and PD-L1-positive exosomes [137]. The key nanotechnologies in this platform were a nanoporous Au nanocluster membrane modified with capture antibodies and a second antibody–conjugated Au nanorod probe, which together enabled highly selective enrichment of cancer-derived vesicles and detection by resonance Rayleigh scattering. The system achieved femtomolar sensitivity (∼10^3^ particles/mL equivalent) and successfully distinguished lung cancer patients from controls, underscoring its non-invasive and clinically relevant design. For HER2-positive breast cancer, a microfluidic chip-based magnetically labeled exosome isolation system (MEIS-chip) was developed [138]. This platform employed antibody-coated MNPs as both capture and manipulation agents for detecting HER2-and CD63-positive exosomes. Within approximately 90 min, it clearly distinguished HER2-positive from HER2-negative patients, demonstrating the role of magnetic nanotechnology in effective exosome isolation for cancer diagnosis and treatment monitoring.

Other systems have been designed to enable multiplex protein and nucleic acid profiling. A filter–electrochemical chip for breast cancer classification integrated nanoporous filtration membranes with electrochemical immunosensors (Fig. 8B) [134]. These nanofilters provided precise size-based isolation, while nanostructured electrodes supported sensitive detection of PMSA-, EGFR-, CD81^−^, and CEA-positive exosomes, with detection limits as low as 1.0 × 10^4^ particles/mL within 100 min, successfully distinguishing breast cancer cases. Another microfluidic device combined immunocapture modules with nucleic acid hybridization assays to simultaneously detect exosomal proteins (EGFR, EpCAM) and miRNAs (miR-21, miR-200) [139]. This platform incorporated nanocomposite-modified capture surfaces and fluorescent probes, enabling isolation and detection of exosomal biomarkers with limits of detection (LODs) of 18, 25, 83, and 35 exosomes/μL for EGFR, EpCAM, miRNA-21, and miRNA-200, respectively. These results allowed the research team to clearly differentiate cancer subtypes, as well as cancer patients from healthy individuals, through quantitative analysis of disease-specific surface proteins and miRNAs derived from both lung cancer cell lines and clinical serum samples.

Integration of on-chip amplification has also been demonstrated. A dual-control microfluidic device combined antibody-functionalized magnetic beads with electrokinetic focusing, followed by dual HCR amplification (Fig. 8C) [135]. Magnetic nanobeads facilitated efficient capture and transport of TDEs, while nucleic acid nanostructures mediated signal amplification. The device detected exosomes with an exceptional LOD of 10.9 particles/μL from 10 μL of serum in 155 min, reliably identifying breast cancer (MCF-7) cells. Similarly, a chip for gastric cancer employed CD66b-coated chambers to capture neutrophil-derived vesicles, followed by rolling circle amplification of miRNAs (Fig. 8D) [136]. Antibody-functionalized nanostructured surfaces improved capture specificity, enabling LODs of 100 copies/μL from 10 μL of serum in under 50 min. With on-chip RCA triggered by aptamers and miRNAs released from heat-lysed neutrophil-derived extracellular vesicles (NEVs), the integrated microfluidic chip (IMCN) detected miR-223–3p and miR-425–5p by fluorescence with LODs of 0.61 and 0.24 fM, respectively, in 4 h.

Finally, nanomaterial-enhanced microfluidic platforms have demonstrated remarkable synergistic performance. For instance, a chip designed for detecting hypoxia-associated miR-210 in breast cancer integrated carbon nanomaterial-coated magnetic beads [139]. These multifunctional beads enabled simultaneous magnetic isolation and FRET-based fluorescence quenching and recovery, achieving a detection limit of 5 pM within 85 min from 1 mL of biofluid. The system successfully distinguished breast cancer patients from healthy controls, exemplifying how multifunctional nanomaterials can streamline the entire exosome analysis workflow.

Collectively, these studies highlight that microfluidic TASs powered by various nanotechnologies such as magnetic nanoparticles, nanoporous membranes, carbon nanomaterials, and hybrid nanocomposites provide the detection of rapid, sensitive, and clinically verified exosome biomarkers. From a design perspective, microfluidic TAS follows the philosophy of miniaturization and automation, providing a rapid, low-capacity, and multiplexed assay that is ideally suited for POC diagnosis. While the integration of magnetic nanomaterials and plasmonic nanomaterials ensures high analytical sensitivity, the reliance on precise microfabrication and pneumatic flow control still limits scalability and mass production.

Several representative microfluidic TAS platforms, such as ExoSearch, iMER, and ExoChip, have already been validated using clinical plasma samples, achieving diagnostic accuracy of over 90 % for breast, lung, and ovarian cancers. In addition to proof-of-concept demonstrations, these systems incorporate semi-automatic operation through integrated pneumatic valves, magnetic microactuators, and multiplexed detection chambers to ensure reproducible analysis in the form of compact cartridges suitable for POC deployments. Nevertheless, obstacles to translation remain, such as the high manufacturing cost of multilayer PDMS chips, limited valve durability, and the need for standardized GMP manufacturing disposable cartridges. Overcoming these engineering barriers is essential to advancing nanomaterial-enabled microfluidic TAS from academic prototypes to scalable clinical implementations.

### 5.2 DISK-type TAS for exosomal biomarker detection

Disk-type TAS, also referred to as centrifugal “lab-on-a-disk” (LOAD) systems, utilize rotational forces to manipulate fluids in a sequential and automated manner, thereby eliminating the need for external pumps or valves. By integrating sample loading, exosome isolation, molecular processing, and detection into a single compact disk, these devices enable true “sample-in, answer-out” workflows. Incorporating nanotechnologies such as nanoporous membranes, immunomagnetic nanoparticles, CRISPR-based nucleic acid reporters, and plasmonic probes further enhances their capacity to achieve sensitive and clinically relevant exosome analysis. Recent advances have shown that disk-type TAS can.

Reliably detect cancer-derived exosomes in patient samples with high diagnostic accuracy.

**Fig. 9:**
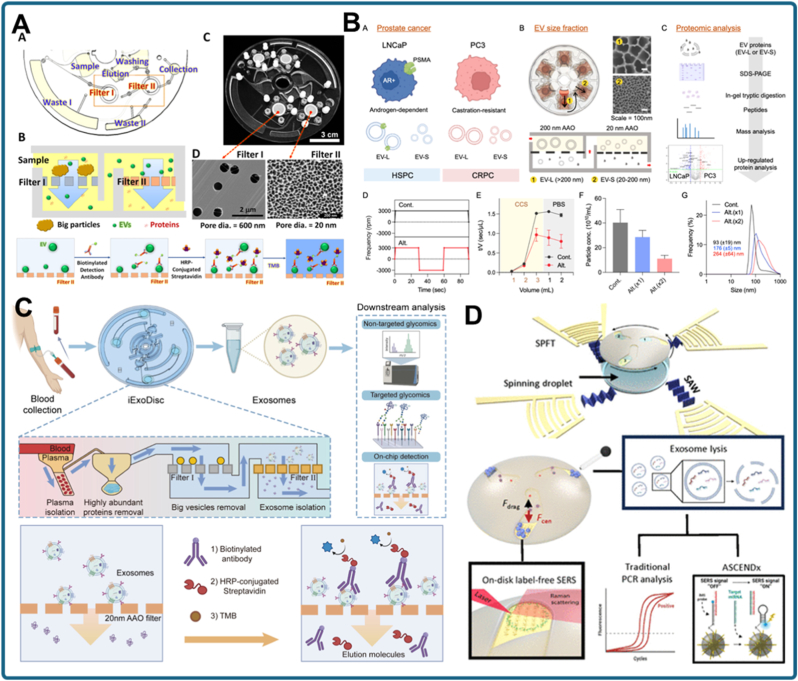
Disk-type TAS platforms for automated isolation and molecular detection of exosomes. (a) Exodisc platform using nanoporous membranes under centrifugal force for rapid, size-selective enrichment of exosomes from clinical plasma and urine samples, enabling bladder cancer diagnosis [140]. (b) Size-dependent separation of exosomes with Exodisc facilitating proteomic analysis in prostate cancer [141]. (c) Fully integrated centrifugal microfluidic disk performing sequential isolation, glycan analysis, and POC diagnosis [142]. (d) ASCENDx system combining surface acoustic wave (SAW) technology with centrifugal fluidics for label-free exosome concentration, followed by isothermal nucleic acid amplification, enabling sensitive detection of colorectal cancer–derived exosomal nucleic acids [143].

One of the most prominent examples is the Exodisc platform, designed for the rapid and size-selective isolation of nanoscale extracellular vesicles (Fig. 9A) [140]. In this system, biological fluids such as plasma or urine are loaded into inlet chambers, and centrifugal force drives them through nanoporous membranes that retain vesicles in the 30–150 nm range while smaller proteins and debris are flushed away. The device integrates sequential washing and elution steps controlled solely by spin-speed adjustments, minimizing nonspecific background. Notably, the enriched vesicles can be directly lysed on-disk for downstream protein or nucleic acid analysis. With recovery efficiencies above 95 % from 100 to 400 μL of clinical samples and a total assay time of 60 min, Exodisc has demonstrated reliable detection of TDEs in bladder cancer patients, underscoring its translational potential as a liquid biopsy tool.

Building on this foundation, an enhanced version of Exodisc enabled size-dependent separation of EV subtypes and comprehensive proteomic profiling in prostate cancer cell lines (LNCaP and PC3) (Fig. 9B) [141]. Using optimized centrifugal fractionation, small EVs (EV-S, 20–200 nm) and large EVs (EV-L, >200 nm) were successfully separated. Subsequent LC-MS/MS-based proteomic analysis revealed that EV protein cargo reflected the molecular characteristics of their parental cells more strongly than size differences, with EV-L showing enriched expression of prostate-specific membrane antigen (PSMA)-associated proteins. These findings indicate that disk-type TAS can provide high-resolution EV isolation and facilitate biomarker discovery in cancer theranostics.

To further extend its functionality, a fully integrated iExoDisc system was developed, combining exosome isolation, glycan analysis, and diagnostic readout in a POC format (Fig. 9C) [142]. Using only 400 μL of plasma or other clinical samples, the system automatically enriches exosomes through nanofilter-based centrifugal microfluidics and directly performs glycan profiling. This on-chip system enables both exosome separation and in situ detection with a total assay time of 2 h. Its one-step, user-friendly workflow exemplifies how disk-type TAS can bridge laboratory-grade EV analysis with clinical POC testing.

Beyond filtration-based Exodisc platforms, alternative centrifugal microfluidic strategies have also been developed. The ASCENDx system integrates acoustic separation with centrifugal disc operation to isolate and concentrate exosomes for nucleic acid detection (Fig. 9D) [143]. By leveraging surface acoustic waves (SAWs) to enhance particle focusing, ASCENDx achieves rapid enrichment of EVs and combines this with a SERS-based inverse molecular sentinel (IMS) DNA probe functionalized on gold nanostars. Applied to colorectal cancer patient samples, ASCENDx demonstrated excellent diagnostic performance with a sensitivity of 95.8 % and specificity of 100 %, highlighting the strong potential of coupling acoustic nanotechnology with centrifugal platforms for clinical liquid biopsy.

In addition to the single-target centrifugal microfluidic strategy, the disk-type TAS platform has evolved to support multiple exosome analysis through nanomaterial-based signal encoding. For example, Ono et al. reported a multiple exosome quantification system based on optical disk technology (ExoCounter), in which exosomes were captured and labeled with several kinds of antibody-bound nanobeads composed of different materials [144]. The platform utilized material-dependent differences in optical diffraction signals, especially pulse amplitudes and polarities, allowing the simultaneous discrimination and quantification of multiple exosome subpopulations within a single disk-based analysis. Through rational selection of polymer-based beads, Au nanobeads, and Ag nanobeads and optical simulation-induced optimization, the system succeeded in simultaneously detecting three representative exosome surface markers (CD147, HER2, CD81) without spectral overlap or cross-interference. Importantly, this disk-type TAS showed high analytical sensitivity and had a detection limit of about 10^2^ of exosome/μL directly from serum samples, highlighting how nanomaterial diversity can be effectively utilized within the centrifugal disk architecture to realize robust, high throughput and multiplexed exosome diagnosis. Taken together, these studies illustrate the versatility of disk-type TAS in exosome analysis. By integrating nanostructured filters, acoustic focusing modules, and plasmonic nanomaterials for signal amplification, these systems enable not only rapid exosome isolation but also advanced molecular characterization, including proteomic, glycomic, and nucleic acid profiling. Their ability to deliver high throughput, reduced assay times, and compatibility with small-volume clinical samples positions disk-type TAS as promising platforms for precision cancer diagnostics and POC applications.

From a design perspective, disk-type TAS embody a centrifugal batch-processing and standardization philosophy, enabling sequential multi-step analysis without external pumps or valves. This design offers excellent reproducibility and medium-throughput performance, making it particularly suitable for centralized clinical laboratories. However, the reliance on precise motor alignment, optical calibration, and rotational control introduces mechanical complexity and cost, which may hinder large-scale adoption in decentralized or resource-limited settings.

Recent centrifugal TAS platforms, including Exodisc and Lab-on-a-DISK, have demonstrated substantial progress toward clinical implementation. These systems have been validated with more than 10 clinical serum and plasma samples, achieving complete exosome isolation and biomarker detection within 30 min and showing excellent batch-to-batch reproducibility. Automated control of fluid movement through pre-programmed spin cycles eliminates manual pipetting, while integrated optical readout modules enable simultaneous detection of multiple biomarkers in a single disk. Collectively, these advances confirm the feasibility of disk-type TAS for semi-automated clinical screening, bridging the gap between point-of-care testing and centralized laboratory workflows. Nevertheless, further translation requires standardization of motor alignment and cartridge fabrication, reduction of per-test cost, and mechanical durability testing under repeated operation to ensure reliable scale-up and regulatory compliance.

### 5.3 Vial-type TAS for exosomal biomarker detection

Unlike microfluidic or disk-based architectures, vial-type TAS perform the entire workflow—exosome capture, nucleic acid or protein analysis, signal amplification, and detection—within a single vial or reaction tube. This streamlined configuration eliminates the need for microfabricated channels or centrifugal actuation while maintaining compatibility with routine laboratory equipment. The incorporation of nanotechnologies has been pivotal in enabling rapid, sensitive, and user-friendly cancer diagnostics. Notably, several vial-based systems have demonstrated clinical utility by successfully distinguishing cancer patients, including those with hematological malignancies, from healthy individuals.

**Fig. 10:**
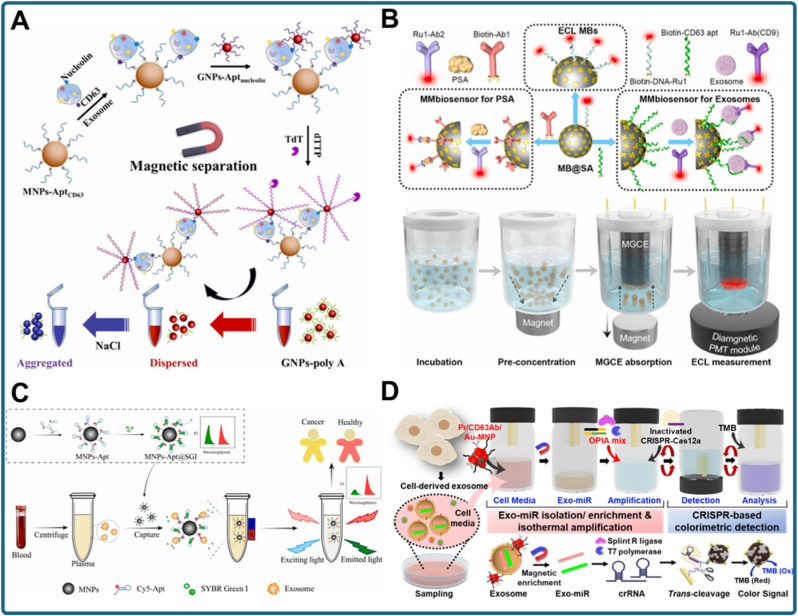
Vial-type total analysis systems (TAS) integrating exosome isolation, amplification, and detection within a single container. (a) Colorimetric aptasensor based on multifunctional spherical nucleic acids (SNAs) and TdT-induced dual signal amplification for the detection of leukemia-derived exosomes, achieving particle-level sensitivity [145,146]. (b) Electrochemiluminescence (ECL) magnetic microbiosensor employing antibody-modified magnetic beads with dual magnetic field actuation to enhance mixing and binding efficiency, enabling sensitive quantification of breast cancer–derived exosomes [147]. (c) One-step, label-free ratiometric fluorescence assay using aggregation-induced emission (AIE) fluorophores and aptamer-functionalized magnetic beads for rapid discrimination of plasma samples from cancer patients [148]. (d) Hourglass-inspired TAS (H-TAS) combining gold–satellite–magnetic nanoparticles for exosome capture and amplification with CRISPR-Cas12a enzyme-encapsulated nanoprobes for colorimetric readout, enabling femtomolar detection of exosomal miR-21 in breast cancer clinical samples [149]

A representative example is a colorimetric aptasensor that integrates multifunctional spherical nucleic acids (SNAs) with terminal deoxynucleotidyl transferase (TdT)-induced dual amplification for leukemia diagnosis (Fig. 10A) [145]. SNAs serve as high-density platforms for aptamer functionalization, enabling dual recognition of leukemia-derived exosomes through CD63 and nucleolin markers. TdT elongates aptamer termini, generating polyT tails that hybridize with polyA-linked AuNPs. Subsequent AuNP aggregation produces a visible color change detectable by the naked eye. Entirely contained within a single vial, this assay achieved a detection limit of approximately 45 particles/μL and clearly distinguished leukemia patients from healthy controls, demonstrating the feasibility of exosome-based liquid biopsy in hematologic cancers.

Another platform employs electrochemiluminescence (ECL) magnetic microbiosensors with dual magnetic field actuation [116]. Antibody-modified magnetic microbeads are manipulated by alternating magnetic fields within a vial to enhance mixing and improve antigen–antibody interactions. Luminophore-conjugated probes generate ECL signals proportional to exosome concentration. By leveraging nanostructured Ru(bpy)_3_^2+^-linked beads, this assay achieved a detection limit of 4.9 × 10^2^ particles/mL and successfully quantified breast cancer-derived exosomes, underscoring how enhanced magnetic actuation can significantly improve assay sensitivity and reproducibility in vial-type formats.

A third study developed a one-step, label-free ratiometric fluorescence assay for plasma exosomes (Fig. 10B) [147]. This platform employed aggregation-induced emission (AIE) fluorophores coupled with aptamer hairpins immobilized on MNPs. Binding of exosomes to CD63-specific aptamers induced conformational changes that released SYBR Green I (SGI), producing a measurable ratiometric fluorescence shift between the Cy5 and SGI channels (the system called MNPs-Apt@SGI). Conducted entirely within a single vial and requiring no labeling or washing steps, the assay delivered results in under 30 min with a detection limit of 4.0 × 10^4^ particles/μL of CD63-positive exosomes. Clinical plasma testing confirmed that the system reliably distinguished lung cancer patients from healthy individuals (AUC = 0.85), underscoring its diagnostic robustness.

Nanotechnology has also enabled spectroscopic analysis of exosomes. A magnetic SERS platform combined antibody-coated Fe_3_O_4_ nanoparticles for exosome capture with SERS-active metallic substrates for downstream molecular fingerprinting (Fig. 10C) [148]. In this system, exosomes from breast cancer patients were first magnetically isolated and then transferred onto SERS substrates for label-free profiling. The platform differentiated breast cancer patients from healthy controls with >90 % diagnostic accuracy, demonstrating how magnetic nanomaterials and plasmonic nanostructures can be seamlessly combined in a vial-based workflow for high-specificity cancer detection.

The hourglass-inspired total analysis system (H-TAS) represents one of the most advanced vial-type TAS, integrating exosome isolation, nucleic acid amplification, and CRISPR-Cas12a detection into a single, one-pot workflow (Fig. 10D) [149]. Conventional CRISPR assays for exosomal miRNA detection are often hampered by multiple steps, including external isolation and complex amplification. H-TAS overcomes these limitations through an innovative hourglass-shaped device that enables all reactions to proceed sequentially with a simple flipping action. The system is divided into two functional modules: (i) exosome capture and one-pot isothermal amplification using promoter/antibody-enriched gold–satellite–MNPs, which simultaneously enrich vesicles and introduce promoter sequences for downstream transcription, and (ii) enhanced colorimetric signal generation via multi-enzyme-encapsulated nanoparticles coupled with CRISPR-Cas12a collateral cleavage. In proof-of-concept experiments, H-TAS targeted exosomal miR-21, achieving an impressive detection limit of 1.308 fM with a total sample-to-answer time of ∼190 min. More importantly, the system was validated not only with breast cancer cell line–derived exosomes but also with clinical patient serum samples, clearly distinguishing cancer patients from healthy controls.

Collectively, these systems demonstrate that vial-type TAS, empowered by diverse nanotechnologies, can achieve clinically relevant sensitivity and specificity across a wide range of cancers. By enabling rapid, one-pot workflows with detection limits down to tens of particles per microliter or femtomolar concentrations, they provide compelling evidence that even the simplest TAS format can reliably translate exosomal biomarkers into actionable diagnostic information.From a design perspective, vial-type TAS implements a simplification and accessibility philosophy that integrates all analytical steps, from exosome isolation and amplification to signal reading, within a single reaction vessel. This independent configuration is particularly suitable for POC diagnostic and resource-limited environments with minimal user intervention and reduced risk of contamination. While the simplicity of the vial-type platform provides good usability and cost-effectiveness, low multiplexing capacity and limited automation module integration remain major constraints in high-throughput clinical applications.

Building on these developments, the vial-type TAS platform has achieved significant clinical validation beyond conceptual demonstrations. For example, H-TAS was tested with 12 clinical samples, successfully distinguishing breast cancer patients from healthy controls [150]. Similarly, MNPs-Apt@SGI-based single vial TAS demonstrates clinical performance by testing 26 clinical samples and successfully distinguishing lung cancer patients from healthy controls. Their one-pot configuration ensures reproducible signal generation while eliminating sample metastasis, reducing contamination and operator dependence. The system demonstrates the practical potential of vial-type TASs for fully independent and cost-effective clinical screening. Nevertheless, for further translation, automatic reagent loading, precision thermal control, and integration of optical or electrochemical reading modules within sealed cartridges will require the realization of operator-independent continuous operation. For large-scale clinical deployment and regulatory approval, establishing multiple normalization modules (internal miRNA-U6, GAPDH) and inter-batch calibration standards are also essential.

Therefore, recent vial-type TAS platforms have further evolved toward simultaneous dual-target detection through integrated amplification and fluorescence-based readout strategies. A representative example is a single-tube TAS that incorporates one-pot miRNA elongation and amplification (OPIEA) to enable ratiometric detection of exosomal miRNAs within a closed reaction vessel [151]. In this system, after nanomaterial-assisted exosome capture, SplintR ligase-mediated elongation of the target miRNA follows, creating an extension template that initiates transcriptional amplification by T7 polymerase under isothermal conditions. This OPIEA process allows direct signal amplification from short exosomal miRNAs without the need for reverse transcription or thermal cycling. Importantly, the amplified RNA products are detected via orthogonal light-up RNA aptamers that selectively bind fluorogenic dyes, resulting in fluorescence emission only upon correct target-dependent transcription. By enabling concurrent fluorescence activation from a cancer-associated miRNA and an endogenous reference miRNA within the same tube, this ratiometric readout effectively compensates for variability in exosome isolation efficiency and input abundance. Also, single-tube TAS demonstrated high sensitivity, with limits of detection of 1.179 fM for miR-21 and 843.9 aM for miR-U6, and high specificity within a short assay time (sample-to-answer = 150 min) with successful application in clinical sample (AUC = 1). Together, the integration of OPIEA-based isothermal amplification and light-up aptamer fluorescence highlights how vial-type TAS platforms can achieve both high analytical sensitivity and internal normalization while maintaining operational simplicity for translational exosome diagnostics.

Taken together, these studies indicate that vial-type TAS platforms have progressed beyond proof-of-concept demonstrations toward clinically relevant, quantitatively reliable diagnostic systems. By integrating nanomaterial-assisted exosome capture, isothermal amplification, and closed-vessel signal readout within a single vial or tube, these platforms effectively minimize operational complexity, contamination risk, and user-dependent variability. Importantly, the incorporation of dual-target detection strategies with internal reference normalization enables robust compensation for sample-to-sample variability, strengthening analytical reliability in clinical settings. Such advances position vial-type TAS as a practical and scalable format for translational exosome diagnostics, while also providing a benchmark for comparison with microfluidic and disk-based TAS architectures.

Table 4 summarizes representative nanomaterial-based TAS platforms developed for exosomal biomarker detection in cancer diagnosis. The table compares different platform types (microfluidic, disk, and vial-based systems) in terms of targeted cancer biomarkers, detection methods, nanomaterials employed, sensitivity, and assay time. Overall, microfluidic TAS excel in integration and rapid analysis, disk-type systems offer user-friendly operation, and vial-type approaches remain versatile for laboratory validation. Across all formats, nanomaterials such as MNPs, AuNPs, and nanoporous structures markedly enhance exosome capture and signal readout, leading to high sensitivity and short assay times. These advances underscore the strong potential of nanomaterial-based TAS for POC cancer diagnostics.

**Table 4:** Summary of nanomaterial-based TAS platforms for exosomal biomarker detection in cancer diagnosis.

TAS Type	Cancer Type	Target Biomarkers	Detection Method	Nanotechnology Employed	LOD	Assay Time	Cancer diagnostic metrics (Specificity, Sensitivity, *p*-value etc.)	Ref.
Microfluidic	Breast cancer	EpCAM, CD49f-positive exosomes	Particle counting + fluorescence	Antibody-functionalized microbead	–	–	Specificity: 90 % Sensitivity: 90 % *p* = 0.001	[133]
	Lung cancer	EGFR, PD-L1-positive exosomes	Resonance Rayleigh scattering	Antibody-coated Au nanorod	1 particle/μL	60 min	*p* = 0.00215	[136]
	Breast cancer	HER2/CD63-positive exosomes	Immunomagnetophoresis	MNPs coated with an antibody	–	90 min	*p* < 0.0001	[138]
	Breast cancer	PSMA, EGFR, CD81, and CEA-positive exosome	Electrochemical (immunoassay)	Immuno-nanoporous filter + nanostructured Zr-MOFs	1.0 × 10^4^ particles/μL	100 min	–	[134]
	Breast cancer	EpCAM, EGFR, miRNA-21, miRNA-200-positive exosomes	Fluorescence (hybridization assay)	Nanocomposite-modified capture surfaces	18, 25, 83, and 35 exosomes/μL	390 min	*p* < 0.001	[152,153]
	Breast cancer	Tumor exosomal miRNAs	Fluorescence (dual HCR amplification)	Magnetic nanobeads + nucleic acid nanostructures	10.9 particles/μL	155 min	–	[135]
	Gastric cancer	miR-223–3p, miR-425–5p	Fluorescence (RCA amplification)	Antibody-coated nanostructured chambers	0.61 fM, 0.24 fM	240 min	Specificity: 84.51 % Sensitivity: 83.58 %	[136]
	Breast cancer	miR-210	Fluorescence (FRET assay)	Carbon nanomaterial-coated magnetic beads	5 pM	85 min	–	[139]
Disk	Bladder cancer	CD9, CD81 protein-positive exosome	Particle counting/Colorimetric	Nanoporous membranes	–	60 min	–	[140]
	Prostate cancer	PSMA-correlated proteins	Proteomic Analysis	Nanosized filters	–	–	*p* < 0.0001	[141]
	Breast cancer	Exosomal glycans	Colorimetric	Nanomembranes	–	120 min	–	[142]
	Colorectal cancer	miR-21, miR-221	SERS	Surface acoustic wave nanodevices	19 pg, 17 pg	30 min	Specificity: 100 % Sensitivity: 95.8 %	[143]
	Breast cancer	CD147 HER-2 CD81	Laser scanning	Optical diffraction probe (Polymer, Au, Ag nanobead)	9.0 × 10^5^ 8.8 × 10^5^ 8.6 × 10^5^ exosomes/μg	280 min	–	[147]
Vial	Leukemia (blood cancer)	Nucleolin-positive exosomes	Colorimetric	Multifunctional SNAs + AuNPs + TdT polymerization	45 particles/μL	60 min	–	[145]
	Prostate cancer	PSA-positive exosomes	Electrochemiluminescence (ECL)	Antibody-modified magnetic beads + Ru(bpy)_3_^2+^ probes	0.49 particles/μL	22 min	–	[147]
	Lung cancer	CD63-positive exosomes	Fluorescence (ratiometric, AIE probe)	AIE fluorophores + aptamer hairpins on magnetic beads	4.0 × 10^4^ particles/μL	30 min	*p* < 0.05 AUC = 0.85	[148]
	Breast cancer	miR-21	Colorimetric (CRISPR-Cas12a + amplification)	Gold–satellite–MNPs + enzyme nanoprobes	1.308 fM	190 min	*p* < 0.001 AUC = 1	[149]
	Breast cancer	CD9-positive exosomes	SERS (spectral fingerprinting)	Fe_3_O_4_ magnetic beads + SERS substrates	–	–	Specificity: 100 % Sensitivity: 91.67 %	[150]
	Breast cancer	miR-21 miR-U6	Fluorescence	Promoter enriched AuNP with CD63 antibody	1.179 fM 843.9 aM	150 min	P < 0.001 AUC = 1	[151]

### 5.4 Comparative evaluation and practical implications

Three representative architectures (microfluidic, disk, and vial TAS) demonstrate complementary strengths suitable for different operational scenarios. These systems occupy a distinct niche in the clinical test spectrum, which is defined not by competing techniques but by analytical throughput, automation levels, and suitability for POC-to-centralized laboratory deployments.

Microfluidic TASs are inherently suitable for miniaturization, automation, and real-time analysis. Their unified channel network enables continuous sample manipulation, precise reagent metering, and multiple detection within sealed cartridges. These features are ideal for POC applications where sample volume is limited and rapid processing is critical. The high surface-to-volume ratio of microchannels improves mass transfer and reduces incubation time, enabling quantitative results to be obtained in 30–60 min. However, microfluidic manufacturing requires a cleanroom process and precise fluid control, and channel contamination or bubble formation can impair long-term stability. Scalability is typically achieved through parallel cartridge production rather than throughput increase in a single device.

Disc-type TAS bridges the gap between portable and high-throughput formats. These platforms leverage centrifugal forces for fluid control to enable multiple steps, such as exosome separation, cleaning, amplification, and detection, to be performed sequentially in a multiple spin cycle without an external pump or valve. Due to their batch processing power and use of standard optical readers, they are suitable for semi-automatic screening in clinical laboratories. Multiple detection can be achieved by spatially separating the reaction chamber or changing its radial position. Nevertheless, disk systems rely on motor rotation and optical alignment to limit their actual portability, with moderate manufacturing costs per disk and sample crosstalk that can occur at high rotational speeds.

Vial-type TAS maintains the simplest configuration that incorporates all analytical steps within a conventional reaction vessel or tube. This design minimizes fluid complexity and is compatible with standard laboratory incubators or heating blocks. Thanks to its simple workflow and reagent compatibility, vial-type systems can be easily implemented in low-resource environments or for verification studies. Recently, designs that combine self-enrichment and isothermal amplification (e.g., H-TAS) have demonstrated one-pot operation with good reproducibility. However, their throughput is limited, and manual pipetting limits automation and multiple capacity. Therefore, vial-type TASs are best suited for single sample testing or rapid pre-screening rather than large-scale diagnosis. Taken together, these characteristics show that each TAS configuration reflects a distinct engineering rationale balancing integration, throughput, and accessibility.

From a design perspective, the three architectures implement a complementary philosophy. Microfluidic TAS follows the philosophy of miniaturization and automation, prioritizing integration and real-time precision for POC diagnosis. Disc-type TAS adopts batch processing and standardization approaches to emphasize throughput and reproducibility for medium-scale clinical laboratory use. On the other hand, vial-type TAS embraces simplification and accessibility philosophy to maximize ease of use and affordability in decentralized or resource-constrained settings. These various design logics reflect how engineering priorities change with diagnostic environments, resource availability, and throughput demand.

These systems, when evaluated side by side, highlight distinct advantages depending on the intended application. Microfluidic systems are superior in integration and automation, disk systems are superior in throughput and moderate portability, and vial systems are superior in simplicity and accessibility. From a clinical translation point of view, microfluidic TAS is most promising for decentralized POC testing, and the disk platform better aligns with centralized hospital laboratories that require moderate scalability. Vial-type TAS provides the most cost-effective solution for initial prototyping or emergency screening, where sophisticated equipment is not available. In terms of multiplexing, microfluidic chips offer the highest spatial resolution and parallelization potential, disk systems enable medium-level multi-chamber detection, and vial systems rely on ratio measurement or colorimetric measurement multiplexing via probe chemistry.

A concise comparison of the three TAS platforms is summarized in Table 5, highlighting their characteristic performance metrics, design philosophy, and clinical applicability. This comparison framework demonstrates how engineering priorities vary depending on the diagnostic context. That is, microfluidic TAS emphasizing automation and analytical precision for POC testing, disk-type TAS balancing throughput and reproducibility in clinical labs, and vial-type TAS emphasizing simplicity and affordability in resource-constrained environments. These differences clarify how TAS architectures can be strategically chosen or hybridized to suit specific translation requirements.

**Table 5:** Comparative summary of TAS architectures for exosomal biomarker analysis.

**TAS Type**	Design Philosophy	**Fluidic Control Mechanism**	**POC Suitability**	**Throughput/Scalability**	**Multiplexing Capability**	**Automation Readiness**	**Scalability Hurdles/Translation Challenges**	**[Ref]**
Microfluidic	Miniaturization + Automation	Active flow (pumps, valves, electro-osmotic drives)	Excellent – portable cartridges, rapid turnaround	Low per device but parallelizable	High – multi-channel arrays, on-chip barcoding	High – full automation achievable	High PDMS fabrication cost; valve reliability; GMP cartridge standardization needed	[[123], [124], [125], [126], [127], [128], [129], [130], [131], [132], [133], [134], [135], [136], [137], [138], [139],^152^]
Disk	Batch Processing + Standardization	Centrifugal (spin-induced radial flow)	Moderate – semi-portable bench devices	Medium (8–24 samples per disk)	Moderate – radial multi-chambers or multi-markers	Medium – semi-automated readout	Motor alignment precision; optical alignment; mold cost for mass production	[[140], [141], [142], [143], [144]]
Vial	Simplification + Accessibility	Passive diffusion/manual pipetting/magnetic enrichment	High – simple and portable	Low (single sample per vial)	Low–Moderate – ratiometric or colorimetric chemistry	Low–Medium – manual to semi-automated	Requires automated reagent dispensing, thermal control, and integrated readout	[145,[147], [148], [149], [150], [151]]

Beyond these design ideas, the actual performance of TAS platforms is largely determined by engineering reliability and signal consistency. The stability of microfluidic valves, optical alignment in disk-type devices, and cross-contamination between reaction chambers critically affect analytical precision [154].

Microfluidic TASs enable multiplex detection through miniaturized channels but require strict valve control and signal isolation. Disk-type systems can process multiple samples simultaneously yet are sensitive to misalignment or vibration during rotation. Vial-type TASs offer simplicity and affordability but are generally limited to single-color readouts within one reaction vessel. To enhance analytical robustness, consistent calibration procedures, on-chip reference standards, and improved fluidic separation designs should be implemented to minimize signal interference and batch variation.

## 6 Challenges and future directions

Despite the significant advances achieved with nanomaterial-based TAS for exosomal biomarker detection, several obstacles must be overcome before these technologies can be translated into reliable clinical diagnostics. One of the most fundamental challenges is the lack of standardization and reproducibility. Current TAS platforms employ a wide range of isolation and detection strategies, including immunoaffinity capture, size-based filtration, and magnetic enrichment, each of which enriches different exosome subpopulations [155]. Additionally, nanomaterials used in these systems often exhibit batch-to-batch variability in synthesis, surface modification, and stability, resulting in inconsistent analytical outcomes across laboratories [156]. Establishing reference standards, harmonized operating procedures, and rigorous quality-control frameworks will therefore be essential to achieve reproducibility and meet regulatory requirements. To address these limitations, future TAS should adopt good manufacturing practice (GMP)-compliant nanomaterial synthesis pipelines combined with automated cartridge fabrication and AI-assisted quality-control analytics capable of tracking batch metadata and predicting performance drift across devices. These efforts will ensure long-term reproducibility and traceability across production lots.

Clinical validation and scalability are another major limitation. Most TAS platforms have been demonstrated at the proof-of-concept stage using spiked samples or small patient cohorts, but robust multicenter clinical trials remain scarce. Without validation across large and diverse populations, diagnostic accuracy cannot be fully established. Moreover, the scalability of TAS for routine use in hospital laboratories is still uncertain. While many platforms can achieve detection limits in the femtomolar range or as low as a few particles per microliter, adapting them to high-throughput workflows capable of processing hundreds of patient samples daily is a formidable challenge. Addressing this will require automation and cost-effective nanomaterial production. Equally important is ensuring compatibility with existing laboratory infrastructure. Developing modular TAS cartridges that can be robotically fabricated and reconfigured for different assays could dramatically improve scalability while maintaining analytical consistency [157]. Integrating magnetic enrichment, plasmonic amplification, and digital microfluidics into such plug-and-play modules may enable flexible, standardized architectures adaptable to both centralized and decentralized testing environments.

The intrinsic biological complexity of exosomes further complicates TAS development. Exosomes are highly heterogeneous in size, cargo, and cellular origin, and their physical properties overlap with those of other extracellular vesicles and lipoproteins [158]. The reliance of many TAS on a limited set of surface markers risks introducing bias toward specific exosome subsets and may overlook clinically relevant populations [157]. For instance, immunomagnetic microfluidic TAS tend to enrich tetraspanin-rich vesicles such as CD9^+^ or CD63^+^ exosomes, whereas centrifugal or nanoporous disk systems recover a broader and more heterogeneous vesicle population that may dilute disease-relevant biomarkers. Consequently, variations in diagnostic signal intensity can arise from the isolation principle itself. This methodological artifact may mask genuine biological differences. To mitigate such isolation bias, combining immunoaffinity and size-based strategies, integrating multi-marker capture layers, or normalizing readouts by particle-to-protein ratios can improve the accuracy of quantitative comparisons among TAS platforms.

Similarly, focusing on a single biomarker class, whether nucleic acids, proteins, or glycan, cannot fully capture the complexity of tumor-derived exosomes [158]. Future systems must therefore evolve toward multiparametric profiling, combining nucleic acid, protein, and glycomic signatures, and leveraging multifunctional nanomaterials capable of simultaneous multi-target capture and detection. Furthermore, incorporating AI-driven data analytics can correlate multi-omic exosomal signatures and extract hidden diagnostic patterns, allowing precise disease stratification and reducing false-positive rates [159]. Such integration of machine learning will enable TAS platforms to shift from single-marker detection to comprehensive, system-level diagnostic interpretation.

In addition to scientific hurdles, translational and regulatory challenges must also be addressed. The incorporation of engineered nanomaterials into diagnostic devices raises concerns about their long-term stability, safety, and environmental impact, even though these platforms operate *ex vivo*. At the same time, the regulatory pathway for nanomaterial-enabled diagnostics remains poorly defined. Approval will require comprehensive evidence of reproducibility, scalability, and cost-effectiveness, as well as demonstration of robust performance in real-world clinical environments. Early collaboration with regulatory bodies and industrial partners will be essential to streamline the approval process and ensure manufacturability under GMP standards. Establishing standardized synthetic reference vesicles with defined size, charge, and epitope profiles, together with digital-twin calibration models, will be critical for quantitative benchmarking across laboratories. Once implemented, these approaches could link raw optical or electrochemical readouts to absolute biomarker concentrations, ensuring comparability and accelerating regulatory approval.

Looking ahead, promising directions for nanomaterial-based TAS include the development of multifunctional nanomaterials that combine capture, amplification, and antifouling properties within a single construct, thereby simplifying device design and minimizing variability. Integrating artificial intelligence and machine learning could transform complex multiplexed exosomal signatures into clinically meaningful diagnostic outputs, improving sensitivity and specificity. Hybrid TAS architectures that merge the strengths of microfluidic, disk, and vial formats may also provide flexible solutions tailored to different diagnostic contexts. Coupling TAS with portable readers, smartphone imaging, or wearable biosensors, together with scalable GMP-grade nanomanufacturing, could extend exosome monitoring beyond hospitals while ensuring reproducible large-scale fabrication. Sustainability-focused design using recyclable polymers, low reagent volumes, and lyophilized reagents will minimize waste and reduce environmental impact. In the long term, building a global standardization and data-sharing network that links TAS outputs with AI-driven clinical databases could transform these systems into intelligent, continuously learning diagnostic ecosystems. Collectively, by merging AI-guided optimization, modular automation, and sustainable nanomanufacturing, next-generation TAS will achieve the precision, reproducibility, and accessibility required for routine exosome-based liquid biopsy and personalized oncology.

## 7 Conclusion

Nanomaterial-based TAS represent a transformative approach to exosomal biomarker detection for cancer diagnosis. By integrating exosome isolation, molecular amplification, and detection into a single platform, these systems address the long-standing fragmentation of analytical workflows that has hindered the clinical application of exosome research. Microfluidic-type TAS enable precise fluid manipulation and multiplex analysis with minimal reagent use, disk-type TAS leverage centrifugal forces for automated sample-to-answer operations without pumps or valves, and vial-type TAS consolidate workflows into one-pot assays well suited for resource-limited settings. Across these diverse formats, nanomaterials are central to achieving sensitive and selective exosome capture, enhancing signal transduction, and amplifying weak biomolecular signatures, thereby lowering detection limits to femtomolar levels or just a few particles per microliter in clinically relevant fluids such as plasma, serum, and urine.

In addition to their analytical capabilities, these platforms highlight the multifunctional potential of nanomaterials. AuNPs, magnetic beads, carbon nanostructures, and MOFs each contribute unique functionalities ranging from immunoaffinity capture to optical and electrochemical amplification. Coupling these materials with advanced molecular tools such as CRISPR-Cas systems, isothermal nucleic acid amplification, and sophisticated fluorescent or Raman probes has further expanded the diagnostic capabilities of TAS. Importantly, several platforms have already shown effectiveness with patient-derived samples, enabling robust discrimination between cancer patients and healthy controls and extending their utility to neurodegenerative and inflammatory diseases. Such findings highlight the broad translational potential of nanomaterial-enabled TAS beyond oncology.

Nevertheless, realizing the full clinical impact of these technologies requires overcoming persistent challenges in standardization, reproducibility, scalability, and regulatory approval, as outlined in the previous section. The biological complexity of exosomes, their overlap with other extracellular vesicles, and the heterogeneity of tumor-derived populations demand multiplexed and integrative analytical approaches. The variability in nanomaterial synthesis and functionalization also calls for rigorous quality control and harmonization across laboratories. In addition, clinical validation in large, diverse patient cohorts remains essential before TAS can be adopted as routine diagnostic tools.

Looking ahead, several developments are likely to shape the future of nanomaterial-based TAS. The creation of multifunctional nanomaterials that combine capture, amplification, antifouling, and multiplexing capabilities will streamline device design and enhance reliability. Integrating artificial intelligence and machine learning will help translate complex exosomal datasets into actionable diagnostic insights. Hybrid TAS architectures that unite the strengths of microfluidic, disk, and vial formats may offer flexible solutions for varied healthcare settings, while pairing TAS with portable readers, smartphone-based detection, or wearable devices could extend their use to POC and even at-home monitoring. Parallel efforts by researchers, clinicians, industry partners, and regulatory agencies will be critical to ensure manufacturability, cost-effectiveness, and compliance with clinical standards.

In conclusion, nanomaterial-based TAS hold exceptional promise for turning exosomal biomarkers into practical tools for precision oncology. By enabling rapid, sensitive, and minimally invasive detection, these platforms have the potential not only to facilitate early cancer diagnosis but also to support real-time monitoring of disease progression and treatment response. With continued interdisciplinary innovation and systematic clinical validation, nanomaterial-enabled TAS are well positioned to transition from proof-of-concept demonstrations to fully integrated diagnostic solutions, bringing exosome-based liquid biopsy closer to routine clinical practice and advancing the next generation of personalized medicine.

## CRediT authorship contribution statement

**Myeong-Jun Lee:** Conceptualization, Investigation, Visualization, Writing – original draft, Writing – review & editing. **Minkyu Shin:** Formal analysis, Investigation, Visualization, Writing – original draft, Writing – review & editing. **Sangeun Lee:** Investigation, Writing – review & editing. **Shan Liu:** Writing – review & editing. **Sang-Nam Lee:** Conceptualization, Investigation, Supervision, Writing – review & editing. **Jeong-Woo Choi:** Conceptualization, Funding acquisition, Investigation, Supervision, Writing – original draft, Writing – review & editing.

## Declaration of competing interest

The authors declare the following financial interests/personal relationships which may be considered as potential competing interests: Jeong-Woo Choi reports financial support was provided by National Research Foundation of Korea. Sang-Nam Lee reports a relationship with Uniance gene that includes: employment. If there are other authors, they declare that they have no known competing financial interests or personal relationships that could have appeared to influence the work reported in this paper.

## Data Availability

No data was used for the research described in the article.
